# Early Prediction of Autistic Spectrum Disorder Using Developmental Surveillance Data

**DOI:** 10.1001/jamanetworkopen.2023.51052

**Published:** 2024-01-10

**Authors:** Guy Amit, Yonatan Bilu, Tamar Sudry, Meytal Avgil Tsadok, Deena R. Zimmerman, Ravit Baruch, Nitsa Kasir, Pinchas Akiva, Yair Sadaka

**Affiliations:** 1 KI Research Institute, Kfar Malal, Israel; 2 TIMNA Initiative–Big Data Platform, Ministry of Health, Jerusalem, Israel; 3 Maternal Child and Adolescent Department, Public Health Directorate, Ministry of Health, Jerusalem, Israel; 4 National Insurance Institute, Jerusalem, Israel; 5 Faculty of Health Sciences, Ben-Gurion University of the Negev, Beer Sheva, Israel

## Abstract

**Importance:**

With the continuous increase in the prevalence of autistic spectrum disorder (ASD), effective early screening is crucial for initiating timely interventions and improving outcomes.

**Objective:**

To develop predictive models for ASD using routinely collected developmental surveillance data and to assess their performance in predicting ASD at different ages and in different clinical scenarios.

**Design, Setting, and Participants:**

This retrospective cohort study used nationwide data of developmental assessments conducted between January 1, 2014, and January 17, 2023, with minimal follow-up of 4 years and outcome collection in March 2023. Data were from a national program of approximately 1000 maternal child health clinics that perform routine developmental surveillance of children from birth to 6 years of age, serving 70% of children in Israel. The study included all children who were assessed at the maternal child health clinics (N = 1 187 397). Children were excluded if they were born at a gestational age of 33 weeks or earlier, had no record of gestational age, or were followed up for less than 4 years without an ASD outcome. The data set was partitioned at random into a development set (80% of the children) and a holdout evaluation set (20% of the children), both with the same prevalence of ASD outcome.

**Exposures:**

For each child, demographic and birth-related covariates were extracted, as were per-visit growth measurements, quantified developmental milestone assessments, and referral summary covariates. Only information that was available before the prediction age was used for training and evaluating the models.

**Main Outcome and Measure:**

The main outcome was eligibility for a governmental disabled child allowance due to ASD, according to administrative data of the National Insurance Institute of Israel. The performance of the models that predict the outcome was evaluated and compared with previous work on the Modified Checklist for Autism in Toddlers (M-CHAT).

**Results:**

The study included 1 187 397 children (610 588 [51.4%] male). The performance of the ASD prediction models improved with prediction age, with fair accuracy already at 12 months of age. A model that combined longitudinal measures of developmental milestone assessments with a minimal set of demographic variables, which was applied at 18 to 24 months of age, achieved an area under the receiver operating characteristic curve of 0.83, with a sensitivity of 45.1% at a specificity of 95.0%. A model using single-visit assessments achieved an area under the receiver operating characteristic curve of 0.81 and a sensitivity of 41.2% at a specificity of 95.0%. The best performing prediction models surpassed the pooled performance of M-CHAT (sensitivity, 40%; specificity, 95%) reported in studies with a similar design.

**Conclusions and Relevance:**

This cohort study found that ASD can be predicted from routine developmental surveillance data at an accuracy surpassing M-CHAT screening. This tool may be seamlessly integrated in the clinical workflow to improve early identification of children who may benefit from timely interventions.

## Key Points

**Question:**

Can early childhood autism screening be accurately performed using an automatic prediction model based on longitudinal data from routine developmental surveillance?

**Findings:**

In this cohort study of 1.2 million children, prediction models achieved high performance in predicting the likelihood of autistic spectrum disorder, using information from routine developmental assessments. Performance was on par with the Modified Checklist for Autism in Toddlers, a popular autism screening tool.

**Meaning:**

The findings of this study suggest that it may be possible to achieve accurate early screening of autism based on routine developmental surveillance, alleviating the need for designated questionnaires.

## Teaser

This cohort study assesses the performance of predictive models for autism spectrum disorder in various time frames and different clinical scenarios.

## Introduction

The worldwide prevalence of autistic spectrum disorder (ASD) has increased significantly during the past 2 decades and is estimated at approximately 1 in 100 children, with large variations among geographic regions.^1^ In 2020, 2.8% of 8-year-old US children were estimated to have ASD.^2^ Early diagnosis of ASD is important for enabling timely interventions that can improve outcomes and allow children to achieve their full potential.^3^ Accordingly, the American Academy of Pediatrics recommends applying continuous developmental surveillance during well-child visits in addition to designated ASD screenings at 18 and 24 months of age.^4^ Nevertheless, the median age of first ASD diagnosis in the US was 49 months in 2020.^2^

Developmental screening uses validated tools with psychometric measures, typically administered through parent-reported questionnaires. One such widely used tool is the Modified Checklist for Autism in Toddlers (M-CHAT)^5^ and its revised version with follow-up, M-CHAT-R/F,^6^ administered between the ages of 16 and 30 months. The former is composed of a 23-item questionnaire, whereas the latter is a 2-stage tool with 20 items and follow-up questions. The reported diagnostic performance of M-CHAT varies considerably, depending on the study design and the evaluated population. In a recent meta-analysis,^7^ the pooled sensitivity and specificity in 6 studies that used prospective case confirmation from medical records were 40% (range, 31%-70%) and 95% (range, 92%-99%), respectively.

Developmental surveillance is a less formal process than screening and is considered in many countries to be an integral part of standard health care practices, particularly during well-child checkups. As such, health care professionals routinely observe and assess children’s social skills, communication abilities, and behavior, using checklists of age-appropriate milestones. However, developmental surveillance is not considered suitable for pinpointing developmental delays or specific ASD symptoms with high precision.^8^

Recently, a new developmental surveillance scale called Tipat Halav Israeli Surveillance was introduced.^9^ The Tipat Halav Israeli Surveillance scale includes attainment norms of 59 milestones that were derived from more than 4.5 million developmental assessments conducted nationally on a diverse, multicultural population. In addition, the Developmental Surveillance Score (DSS) was devised; this score translates the milestone-based surveillance into a quantitative score that conveys the child’s developmental status during a specified period.^10^

The motivation for the current study is to extend these tools into a new ASD assessment method that can be seamlessly integrated in the clinical workflow to predict ASD likelihood from the data already collected in the routine surveillance process, unlike traditional questionnaire-based screening tools. In addition, we suggest ways to apply this tool in settings without routine longitudinal development surveillance.

Previous work on autism prediction using electronic health records (EHRs) identified covariates that are associated with autism, such as low birth weight, small for gestational age, low Apgar scores,^11^ and other perinatal complications.^12^ More recently, Engelhard et al^13^ reported high predictive value of their EHR-based autism prediction model, applied before 1 year of age. Similarly, Onishchenko et al^14^ used medical claims data to estimate an autism comorbid risk score that aimed to improve the accuracy of M-CHAT. These studies used information about comorbidities and medical procedures that are more frequent in children later diagnosed with ASD. By contrast, we aimed to address early identification of ASD in children who were seemingly healthy, using their developmental surveillance measures rather than their entire medical history. Specifically, we aimed to develop predictive models for ASD and assess their performance in various time frames and different clinical scenarios. We compared these models with M-CHAT to assess the potential benefit of incorporating them in the clinical workflow of ASD screening.

## Methods

In this cohort study, all analyses were performed in accordance with relevant guidelines and regulations. The study protocol was approved by the Soroka University Medical Center institutional ethics committee. The need for informed consent was waived by the Soroka University Medical Center institutional ethics committee owing to the use of deidentified data. This study follows the Strengthening the Reporting of Observational Studies in Epidemiology (STROBE) reporting guidelines for cohort studies.

### Developmental Surveillance in Israel

Developmental surveillance in Israel is performed routinely from birth to 6 years of age, according to national standards, by certified public health nurses in approximately 1000 maternal child health clinics (MCHCs). The assessments include 59 milestones across 4 domains: personal-social, language, fine motor, and gross motor (eTable 7 in Supplement 1).^15^ Parents are instructed to visit an MCHC after postpartum hospital discharge and then when their child is aged 1, 2, 4, 6, 9, 12, 18, 24, 36, 48, and 60 months. Adherence with these visits is high, especially during the first 2 years of life, when the visits coincide with vaccinations. At each visit, age-appropriate milestones are evaluated according to the expected development at that age (denoted *age step*).

Milestone attainment is reported as observed in the clinic, although in cases of lack of cooperation of the child, attainments may be documented according to parental report (approximately 10%). If the evaluated milestone was not attained by either observation or parental report, it is documented as unattained. When a milestone is unattained at an age that exceeds the national norms for 95% of the population,^16^ the child will be referred to a pediatrician for further evaluation of a possible developmental delay.

### Autism Screening and Diagnosis in Israel

Referrals for suspected communication disorders primarily rely on subjective impressions from MCHC nurses, families, and other caregivers. According to the national guidelines, an autism diagnosis must fulfill specific conditions.^17^ The diagnosis must be conducted by a developmental physician or a child psychiatrist and by a psychologist with relevant training. It must adhere to the criteria established in the *Diagnostic and Statistical Manual of Mental Disorders* (Fifth Edition) and include a functional evaluation using Adaptive Behavior Assessment System II or Vineland II tests and developmental/cognitive assessment using the Mullen or Bayley test.^18^ Additionally, autism symptom assessment requires the inclusion of parental and teachers’ questionnaires.

### Developmental Surveillance Data Set

Data from approximately 70% of the children visiting the MCHCs are documented within a single EHR system. The data contain sociodemographic and birth-related information, per-visit information about attainment of developmental milestones, growth measures, concern for the child’s development raised by a nurse or parent, indications of referrals for further evaluation, and developmental tracking programs.

### Outcome Definition

The National Insurance Institute of Israel (NII) is the governmental agency in charge of social security and welfare. It particularly provides a disabled child allowance for children with ASD. Eligible children are also entitled to educational and therapeutic support, incentivizing high use of this resource. Records of children born since January 1, 2014, who received allowance due to ASD on the outcome collection date (March 19, 2023), were obtained from the NII and anonymously linked to the developmental surveillance records. This process provided a binary ASD outcome for each child along with the age at which each child with ASD became eligible.

### Study Cohorts

The cohort included all children born between January 1, 2014, and January 17, 2023, who were followed up at the MCHCs. We used self-reported cultural groups of the participants to demonstrate that the study cohort is representative of the national population and to assess the performance of the prediction models among cultural subgroups. We excluded preterm children born at a gestational age of 33 weeks or earlier (n = 21 975) or with unknown gestational age (n = 25 720). Children who were younger than 4 years on the outcome collection date were excluded unless they already had an ASD outcome (472 466 excluded and 2938 included). We also excluded children with missing information, depending on each model’s specific set of variables and the age range of the prediction. Consequently, each model was based on a cohort of a different size (eTable 2 in Supplement 1). In sensitivity analyses, we also evaluated subgroups of children excluded from the main analysis, such as preterm-born children and children with missing visits.

### Study Design

**Figure 1.**
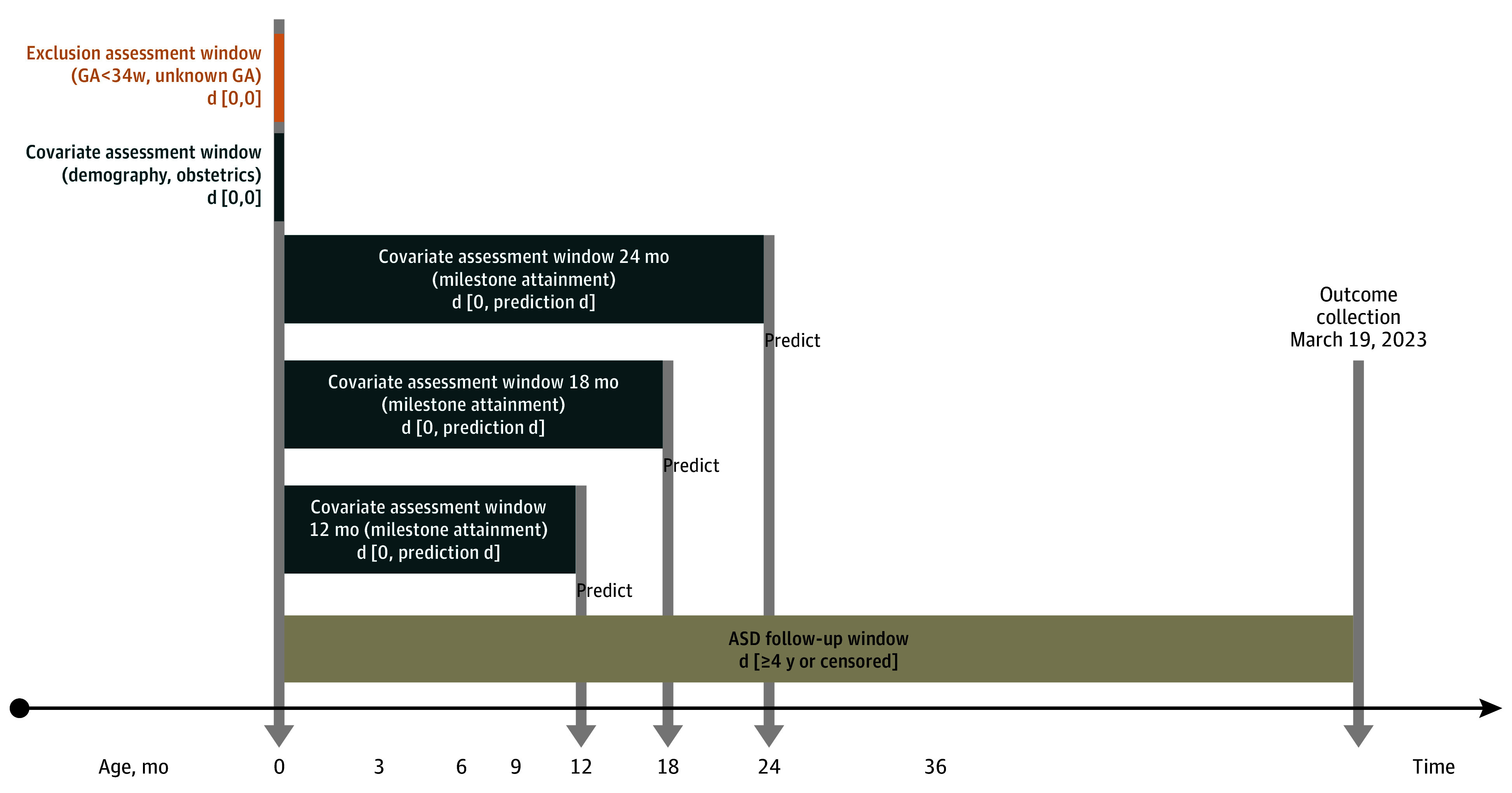
Study Design Birth date is defined as the index date. Several prediction ages are considered, with corresponding covariate assessment windows. Outcome is defined as the child’s status at collection date. ASD indicates autism spectrum disorder; GA, gestational age.

This is a retrospective cohort study, with the child’s birth date set as the index date (Figure 1). We defined 6 covariate assessment windows, starting at birth and ending at 6, 9, 12, 18, 24, and 36 months of age. Prediction of ASD was evaluated at each of these ages, using the covariates available in the corresponding time window. Binary outcome of ASD diagnosis was determined for all children on March 19, 2023, based on eligibility of NII allowance. Because the developmental surveillance process is performed routinely for all children, independent of the outcome information, our study design treats the ASD outcome as a binary variable determined on the outcome collection date, regardless of the age of eligibility, which may precede the prediction age.

### Prediction Models

For each child, we extracted multiple variables, including maternal demography, birth-related measures, longitudinal growth measures, and longitudinal developmental assessments, quantified as milestone scores. The full details of the extracted features and the milestone scores are given in the eMethods in Supplement 1. The models were trained using a development set (80% of the children) and evaluated using a holdout evaluation set (20% of the children); the full details of the training and evaluation process are given in the eMethods in Supplement 1.

We defined 6 types of models, which differ by their sets of variables: a full model, compact model, snapshot-score model, snapshot-binary model, system model, and demographics model. The full model included all extracted variables, including demographic, birth related, aggregated milestone scores, growth measures, and visit summaries. The compact model included a minimal subset of variables that provided performance on par with the full set. This model comprised milestone scores, child’s sex, mother’s age, and maternal educational level. The snapshot-score model included the same variables as the compact set, but the milestone scores were restricted to a single age step (corresponding to the specific prediction age), which aims to emulate a clinical scenario of a single well-child visit rather than a longitudinal evaluation. The snapshot-binary model was the same as the snapshot-score model, except that milestone attainments were encoded as binary passed or failed rather than as a continuous score. This model evaluates a simplified score, which is independent of a specific developmental scale. The system model included variables reflecting the summary reports of the clinical surveillance process: concern for the child’s development or growth raised by the parents or the nurse, referral for further consideration by a pediatrician, and participation in a developmental tracking program, along with demographic and birth-related variables. The demographics model included a baseline set of variables, representing the demographic and birth-related information.

We examined 6 possible age ranges for the prediction: 0 to 6, 6 to 9, 9 to 12, 12 to 18, 18 to 24, and 24 to 36 months, where each age range includes the lower limit and excludes the upper limit (for example, the 24- to 36-month range refers to ≥24 to <36 months). Each prediction model was defined by a combination of variables and a prediction age range. Sensitivity analyses of assessing the models on subgroups of children were conducted as described in the eMethods in Supplement 1.

### Performance Measures

To evaluate the prediction models, we used standard measures of diagnostic performance, including area under the receiver operating characteristic curve (AUC), sensitivity, specificity, and positive predictive value (PPV). To compare models, PPV was measured in the children with the highest *k* percent of the predicted ASD score (denoted PPV at *k*), and sensitivity was measured at a fixed specificity level (typically 95%). When comparing to studies reporting the performance of M-CHAT, we set the threshold on the predicted score to obtain the reported specificity and compared the sensitivities.

### Statistical Analysis

We computed the crude (unadjusted) odds ratio of the ASD outcome for each single variable, along with 95% CIs. For categorical variables, the most common category was taken as a reference. Continuous variables were converted into categorical by data binning. For milestone attainment covariates, we defined a variable indicating any failure at a task from a given age step and a given domain.

Confidence intervals of the prediction performance measures were estimated using bootstrapping with 1000 iterations. To assess the significance of the differences between models, we compared their sensitivities with the McNemar test and their AUC using the DeLong test.^19^ For assessing differences between subgroups, AUCs were compared using Hanley and McNeil’s method,^20^ and sensitivities were compared by applying a χ^2^ test. Comparisons to the sensitivities reported for M-CHAT were done using a χ^2^ test. A 2-sided *P* < .05 was considered to be statistically significant. All analyses were coded using Python, version 3.9.13 (Python Software Foundation). Statistical analysis was done using statsmodels, version 0.13.2. Prediction models were constructed and assessed using xgboost, version 1.5.0 and scikit-learn, version 1.0.2.

## Results

### Population Characteristics

The data set included 1 187 397 children (610 588 [51.4%] male and 576 809 [48.6%] female; 19 573 [1.6%] Druse, 694 460 [58.5%] Jewish, 229 783 [19.4%] Muslim Arab, 20 642 [1.7%] Muslim Bedouin, 37 073 [3.1%] other [Christian Arab, Circassian, other Christian, and other Muslim], and 185 866 [15.7%] missing cultural group data) with a mean (SD) of 5.9 (2.4) MCHC visits per child during the entire observation period and a median (IQR) follow-up time of 709 (445-927) days. The ASD outcome prevalence was 1.55%. Table 1 gives the cohort’s main characteristics. Among children with ASD, compared with those without ASD, there were higher percentages of male children, Jewish children, children of immigrant mothers, children of older (≥40 years) mothers, children with low Apgar scores, and children born by cesarean delivery. eTable 1 in Supplement 1 gives the population characteristics separately for the development and evaluation sets (comprising 949 917 and 237 480 children, respectively), demonstrating that the 2 randomly split sets are similar. The final sizes of the data sets used for training and testing the prediction models are given in eTable 2 in Supplement 1, and the characteristics of the evaluation set before and after exclusions are given in eTable 3 in Supplement 1.

**Table 1.** Characteristics of the Study Participants^a^

Characteristic	All (N = 1 187 397)	No ASD (1 168 992)	ASD (n = 18 405)
Sex			
Female	576 809 (48.6)	572 402 (49.0)	4407 (23.9)
Male	610 588 (51.4)	596 590 (51.0)	13 998 (76.1)
Cultural group			
Druse	19 573 (1.6)	19 473 (1.7)	100 (0.5)
Jewish	694 460 (58.5)	682 354 (58.4)	12 106 (65.8)
Muslim Arab	229 783 (19.4)	228 183 (19.5)	1600 (8.7)
Muslim Bedouin	20 642 (1.7)	20 594 (1.8)	48 (0.3)
Missing	185 866 (15.7)	182 373 (15.6)	3493 (19.0)
Other^b^	37 073 (3.1)	36 015 (3.1)	1058 (5.7)
Mother’s birth country or region			
Europe	20 332 (1.7)	20 115 (1.7)	217 (1.2)
Ethiopia	16 800 (1.4)	16 209 (1.4)	591 (3.2)
Former Soviet Union	63 557 (5.4)	61 281 (5.2)	2276 (12.4)
Israel	919 439 (77.4)	907 115 (77.6)	12 324 (67.0)
North America	13 204 (1.1)	13 067 (1.1)	137 (0.7)
Missing	141 775 (11.9)	139 169 (11.9)	2606 (14.2)
Other^c^	12 290 (1.0)	12 036 (1.0)	254 (1.4)
Employment status			
Not working	228 933 (19.3)	225 686 (19.3)	3247 (17.6)
Student	48 250 (4.1)	47 824 (4.1)	426 (2.3)
Working	521 114 (43.9)	513 127 (43.9)	7987 (43.4)
Missing	389 100 (32.8)	382 355 (32.7)	6745 (36.6)
Mother’s educational level			
Academic	340 480 (28.7)	335 898 (28.7)	4582 (24.9)
Elementary	23 583 (2.0)	23 289 (2.0)	294 (1.6)
High school	292 590 (24.6)	287 301 (24.6)	5289 (28.7)
Tertiary education	118 979 (10.0)	117 452 (10.0)	1527 (8.3)
Missing	411 765 (34.7)	405 052 (34.6)	6713 (36.5)
Mother’s family status			
Divorced	11 636 (1.0)	11 152 (1.0)	484 (2.6)
Married	977 971 (82.4)	964 260 (82.5)	13 711 (74.5)
Widower	580 (0.0)	565 (0.0)	15 (0.1)
Missing	146 379 (12.3)	143 859 (12.3)	2520 (13.7)
Other	50 831 (4.3)	49 156 (4.2)	1675 (9.1)
Consanguinity			
No	335 806 (28.3)	331 457 (28.4)	4349 (23.6)
Yes	66 638 (5.6)	66 169 (5.7)	469 (2.5)
Missing	784 953 (66.1)	771 366 (66.0)	13 587 (73.8)
Length of pregnancy, median (IQR), wk	39.3 (38.3-40.2)	39.3 (38.3-40.2)	39.1 (38.0-40.1)
Birth weight, mean (SD), kg	3.2 (0.5)	3.2 (0.5)	3.2 (0.6)
Head circumference, mean (SD), cm	34.2 (1.6)	34.2 (1.6)	34.2 (1.8)
Apgar score at 1 min			
<8	46 264 (3.9)	45 166 (3.9)	1098 (6.0)
≥8	1 088 400 (91.7)	1 071 957 (91.7)	16 443 (89.3)
Missing	52 733 (4.4)	51 869 (4.4)	864 (4.7)
Apgar score at 5 min			
<8	10 270 (0.9)	10 001 (0.9)	269 (1.5)
≥8	1 116 765 (94.1)	1 099 585 (94.1)	17 180 (93.3)
Missing	60 362 (5.1)	59 406 (5.1)	956 (5.2)
Type of birth			
Cesarean	206 788 (17.4)	202 143 (17.3)	4645 (25.2)
Instrumental	60 714 (5.1)	59 623 (5.1)	1091 (5.9)
Spontaneous	846 135 (71.3)	834 840 (71.4)	11 295 (61.4)
Missing	73 760 (6.2)	72 386 (6.2)	1374 (7.5)
Newborn position			
Breech	38 190 (3.2)	37 339 (3.2)	851 (4.6)
Head	965 618 (81.3)	951 633 (81.4)	13 985 (76.0)
Missing	168 499 (14.2)	165 216 (14.1)	3283 (17.8)
Other	15 090 (1.3)	14 804 (1.3)	286 (1.6)
Mother’s age, y			
≤20	42 114 (3.5)	41 563 (3.6)	551 (3.0)
>20-≤40	1 028 945 (86.7)	1 013 502 (86.7)	15 443 (83.9)
>40	36 053 (3.0)	35 152 (3.0)	901 (4.9)
Missing	80 285 (6.8)	78 775 (6.7)	1510 (8.2)

^a^ Data are presented as number (percentage) of study participants unless otherwise indicated.

^b^ Other includes Christian Arab, Circassian, other Christian, and other Muslim.

^c^ Other includes Africa, Asia, Australia and Oceania, and South and Central America.

### Evaluation of Prediction Models

**Figure 2.**
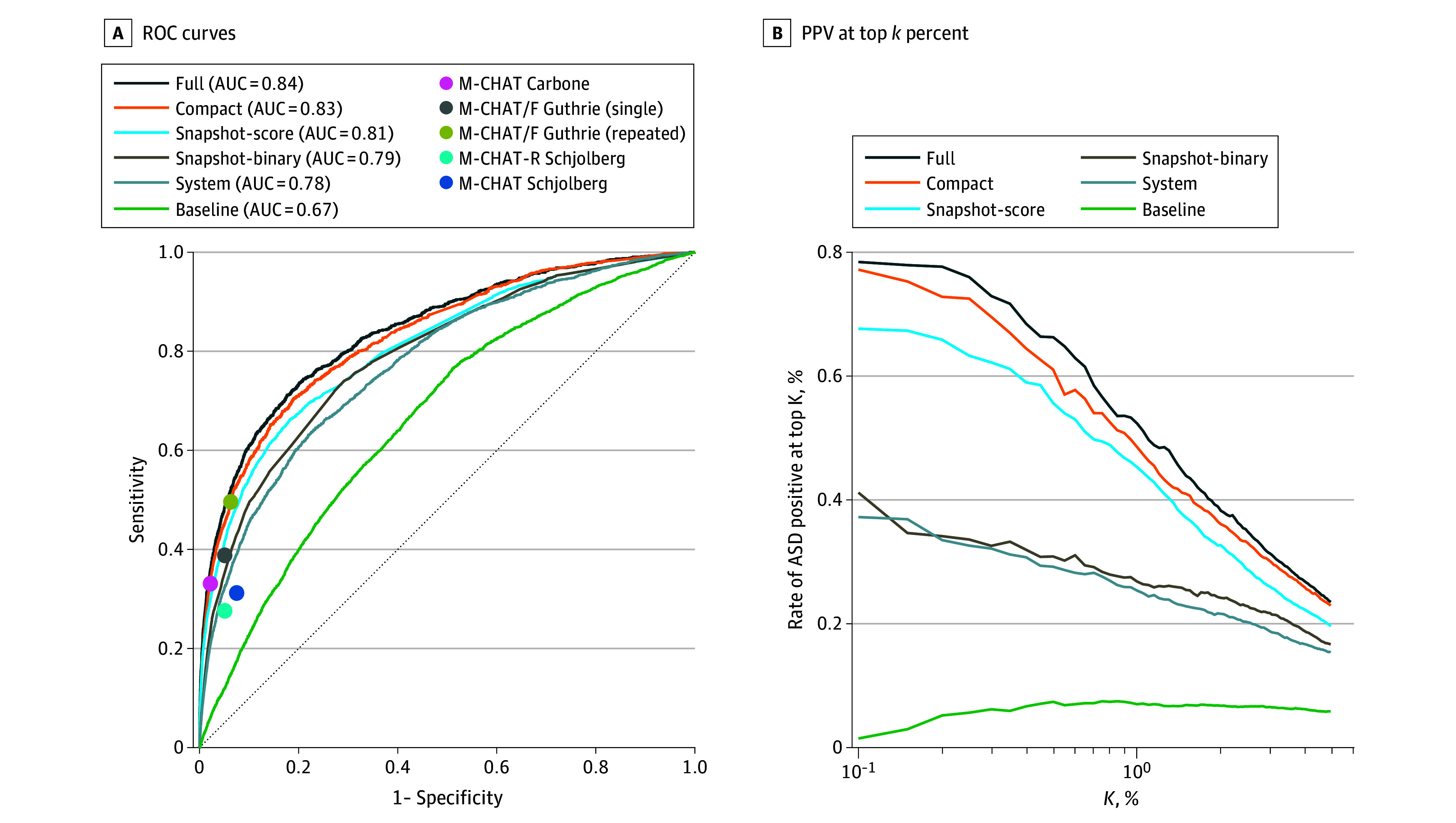
Diagnostic Performance of Prediction Models With Different Sets of Variables Performance of the 6 prediction models on the evaluation set at 24 months of age. A, Receiver operating characteristic curves with the area under the curve (AUC) denoted in the legend, alongside sensitivity and specificity points reported for the Modified Checklist for Autism in Toddlers (M-CHAT) by 3 large prospective studies.^21,22,23^ B, Fraction of children with an autism spectrum disorder (ASD) outcome among those with the highest predicted score by each of the models. The threshold (x-axis) is defined by the top *k* percentage of predicted scores.

Figure 2A depicts receiver operating characteristic curves and the resultant AUC on the evaluation set for each of the model types, with ASD predicted at 24 months of age. eTable 4 in Supplement 1 provides all compared performance measures. The full model had the highest AUC of 0.84, with a sensitivity of 48.1% at a specificity of 95.0%, marginally better than the compact model (AUC, 0.83; sensitivity, 45.1%; *P* < .001 vs the complete model). The snapshot models attained marginally lower AUC (0.81 and 0.79 for the snapshot-score and snapshot-binary models, respectively). The snapshot-score model was significantly more sensitive than the snapshot-binary model at high specificity (41.2% vs 34.4%, *P* < .001). This difference is also apparent in Figure 2B, which depicts the positive predictive value (PPV) when considering the *k* percentage of children with the highest predicted score. The PPV at *k* = 0.1% was 78.3% for the full model, 75.1% for the compact model, 67.8% for the snapshot-score model, and 41.8% for the snapshot-binary model. The system model achieved lower performance than the former models (AUC, 0.78; sensitivity, 32.1%; PPV at *k* = 0.1%, 37.5%; *P* < .001 vs the compact model) but was still considerably better than the baseline demographics model (AUC, 0.67; sensitivity, 11.8%; PPV at *k* = 0.1%, 1.5%; *P* < .001 vs the system model). eTable 4 in Supplement 1 gives the model comparison for prediction at 18 months of age, indicating an AUC decrease of 0.05 to 0.07 and sensitivity decrease of 10.5 to 17.5 percentage points compared with prediction at 24 months of age.

**Figure 3.**
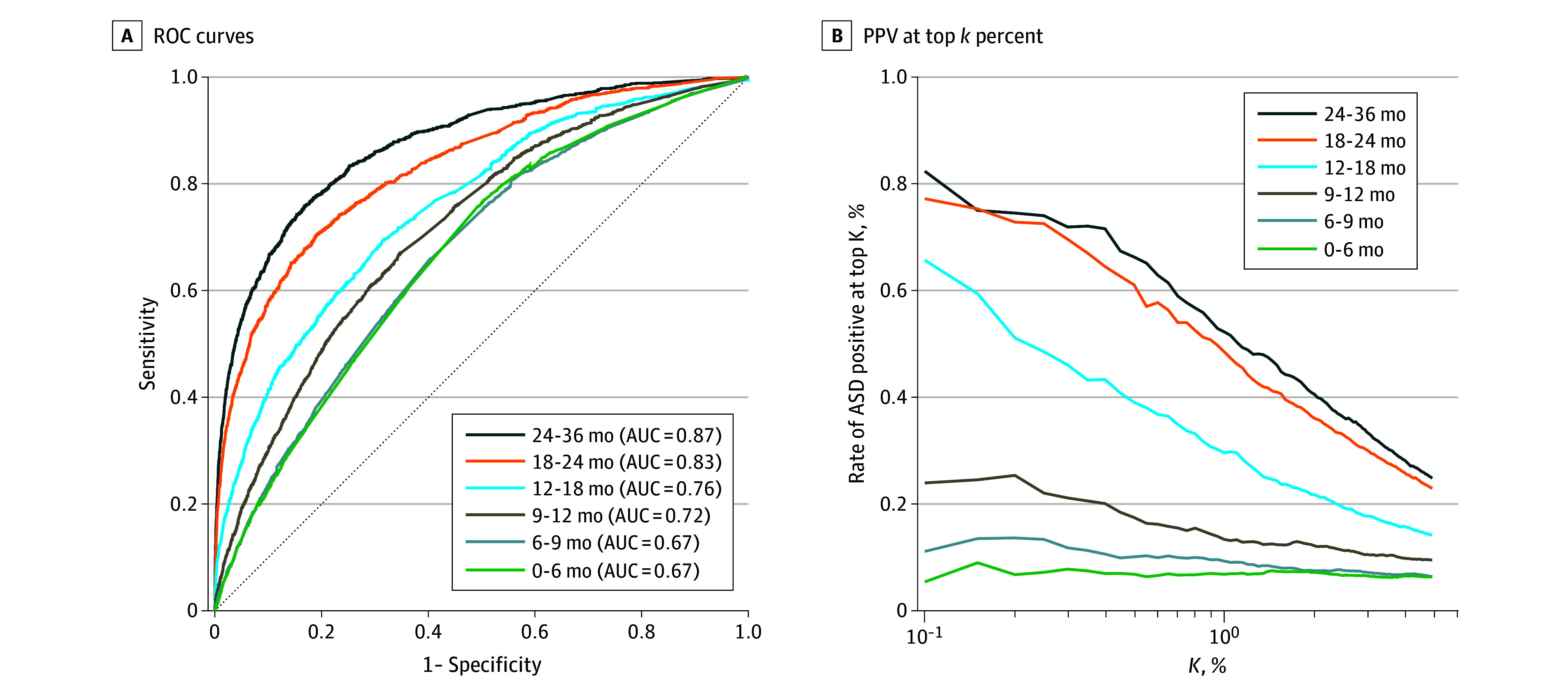
Diagnostic Performance of the Compact Prediction Model at Different Ages Performance of the compact model at different prediction ages. A, Receiver operating characteristic curves with the area under the curve (AUC) denoted in the legend. B, Fraction of children with an autism spectrum disorder outcome among those with the highest predicted score. The threshold (x-axis) is defined by the top *k* percentage of predicted scores.

eTable 5 in Supplement 1 compares the performance at different prediction ages for the compact and the snapshot-score models. Figure 3A depicts this comparison for the compact model. Models based on data available at 6 or 9 months performed similarly and yielded AUC of 0.67 to 0.68, similarly to the baseline demographics model. The performance improves as the child ages as more information is available for the predictor, outperforming the baseline model at 12 months of age (AUC, 0.72 and 0.71 for the compact and snapshot-score models, respectively, vs 0.67 for the baseline model). The compact model, evaluated at 36 months of age, achieved an AUC of 0.87 (*P* < .001 vs the 24-month model), a sensitivity of 54.6% at a specificity of 95.0% (*P* = .28 vs the 24-month model), and a PPV at *k* = 0.1% of 82.0% (Figure 3B). Among the top 0.1% of children with highest predicted score at 12 months of age, 24.7% indeed had ASD, and this measure increased to 65.1% and 75.1% for predictions at 18 and 24 months of age, respectively. Similarly, the snapshot-score model at 36 months achieved an AUC of 0.83, a sensitivity of 47.5%, and a PPV at *k* = 0.1% of 80.4%.

eFigure 1 in Supplement 1 depicts the calibration plots for the 3 best models at the prediction age of 24 months, indicating that the models appear to be well calibrated. The eResults and eFigure 2 in Supplement 1 give the crude odds ratio for an ASD outcome for each of the features.

### Comparison With M-CHAT’s Performance

Figure 2A compares the sensitivity of the ASD prediction models at 24 months of age with the reported performance of M-CHAT and its variants in 3 large retrospective cohort studies.^21,22,23^ eTable 6 in Supplement 1 provides a detailed comparison for the compact model. In each comparison, the ASD score threshold was set to obtain the reported reference specificity. Carbone et al^21^ reported a sensitivity and specificity of 33.1% and 97.8%, respectively, for M-CHAT in a cohort of 26 364 children. Guthrie et al^22^ reported a sensitivity of 38.8% and a specificity of 94.9% (or 49.6% and 93.7%, respectively, when combining repeated screening^7^) for M-CHAT/F in a cohort of 20 375 children. Schjølberg et al^23^ analyzed examinations of 54 463 children to report sensitivities of 31.2% and 27.6% with specificities of 92.5% and 94.9% for M-CHAT and M-CHAT-R, respectively. The sensitivity of our model was higher than or equivalent to the compared references.

### Subgroup Analysis

Table 2 lists the performance of the compact model (with prediction at 24 months of age) on subgroups selected from the evaluation set. Comparison of the performance on demographic subgroups showed that the AUC and the sensitivity were marginally higher for female children than for male children (AUC, 0.83 vs 0.79, *P* = .04; sensitivity, 47.7% vs 42.5%; *P* = .10) and the performance in Arab and Jewish children, the 2 major cultural groups, were similar (AUC, 0.82 vs 0.83, *P* = .69; sensitivity, 43.2% vs 46.0%; *P* = .44). Because the prevalence of ASD is significantly lower for female and Arab infants, the use of a single global threshold for all subgroups decreased sensitivity and increased specificity in the underrepresented subgroups, indicating that group-specific thresholds should be preferred. Similarly, in the subgroup of late preterm-born children, which has higher ASD prevalence than term-born children, the group-specific threshold provided comparable performance (AUC, 0.82 vs 0.83; sensitivity, 42.6% vs 45.0%; *P* = .64), whereas the global threshold increased the sensitivity on the account of specificity.

**Table 2.** Results of the Subgroup Analyses for the Compact Model Applied at 24 Months of Age

Subgroup	No.	Prevalence, %	Using a group-specific threshold^a^		Using a global threshold of all (reference)^b^	
			AUC (95% CI)	Sensitivity at a specificity of 95.0%	Sensitivity, %	Specificity, %
All (reference)	57 190	2.7	0.83 (0.82-0.84)	45.1 (42.6-47.5)	45.1	95.0
Male children	29 353	4.1	0.79 (0.78-0.81)	42.5 (39.6-45.4)	49.7	91.6
Female children	27 837	1.3	0.83 (0.80-0.85)	47.7 (42.5-53.2)	29.6	98.4
Jewish children	28 883	3.4	0.83 (0.82-0.84)	46.0 (42.5-49.2)	47.9	94.3
Arab children	18 563	1.2	0.82 (0.79-0.85)	43.2 (36.9-49.7)	36.0	97.2
Term children	53 705	2.6	0.83 (0.82-0.84)	45.0 (42.3-47.8)	44.4	95.2
Preterm (≥34 wk) children	3485	4.0	0.82 (0.78-0.86)	42.6 (34.6-51.7)	51.8	92.1
No parental concern	52 959	2.0	0.79 (0.78-0.81)	36.1 (33.0-39.0)	31.6	96.1
No nurse concern	51 838	2.1	0.79 (0.78-0.81)	36.7 (33.8-39.7)	30.9	96.3
No referrals	52 381	2.1	0.80 (0.78-0.81)	37.3 (34.5-40.8)	31.9	96.3
No developmental tracking	55 618	2.4	0.82 (0.81-0.83)	42.1 (39.4-45.1)	40.1	95.4
No early concern (any)	47 190	1.6	0.75 (0.74-0.77)	28.4 (25.0-31.7)	18.7	97.2
Missed visit	107 352	2.7	0.81 (0.80-0.81)	40.5 (38.7-42.4)	40.3	95.2
Born before March 19, 2019 (4-y follow-up)	56 969	2.3	0.82 (0.81-0.83)	41.8 (39.4-44.5)	41.8	95.0

^a^ Area under the receiver operating characteristic curve (AUC) and sensitivity at a 95.0% specificity are given with their 95% CIs, assuming the score’s thresholds are group specific.

^b^ Sensitivity and specificity are also given for global score thresholds, derived from the entire evaluation set.

Another subgroup type comprises children without clinical concern for their development at the time of the prediction. These subgroups may include children without a concern raised by the parents or the MCHC nurse or those not referred for further consideration or to a developmental tracking program. We evaluated each of these conditions separately and in combination. The prevalence of ASD outcome in these subgroups was lower than in the entire evaluation set. In all subgroups, the AUC and the sensitivity decreased vs the entire evaluation set (AUC, 0.75-0.82 vs 0.83; sensitivity, 28.4%-42.1% vs 45.1%).

Finally, we examined the model on children who were excluded from the cohort because they were missing up to 4 milestone assessments in any visit or domain. This experiment led to a modest performance degradation with AUC of 0.81 (vs 0.83) and a sensitivity of 40.5% (vs 45.1%).

## Discussion

Early identification and intervention can improve outcomes of children with ASD.^24,25^ We presented a framework for predicting ASD based on data from routine developmental surveillance. Our models achieved good performance for children as young as 12 months of age, with considerable improvement at older ages. We examined several types of models, which differ in their predictive variables. A compact model, which relies mostly on milestone attainment data, is suggested for a clinical scenario of routine longitudinal follow-up, such as the Israeli developmental surveillance program. For a setting of a single well-child visit, a snapshot model, using attainments of age-appropriate milestones, is suggested. Additional models were evaluated for reference: a full model considering all available variables and a demographics-only baseline model.

The results suggest that milestone attainment scores are strong predictors of ASD, and their combination in the compact model achieved similar accuracy to the full model (AUC, 0.83 vs 0.84), with notably fewer variables. The models’ performance improved at older prediction age, outperforming the baseline model at age 12 months (AUC, 0.72 vs 0.67) and reaching high accuracy (AUC, 0.87; sensitivity, 54.6% at a specificity of 95.0%) at 36 months of age. Similarly, among the top 0.1% of children with highest predicted score at 12 months of age, 24.7% indeed had ASD, and this measure increased to 65.1%, 75.1%, and 82.0% for predictions at 18, 24, and 36 months of age, respectively.

The snapshot models were designed for the common setting in which ASD screening is performed in a single well-child visit. Although slightly less accurate than longitudinal assessment, the performance of the snapshot-score model remains high, with an AUC of 0.81 (vs 0.83) and a sensitivity of 41.2% (vs 45.1%) at 24 months of age. Furthermore, when binary milestone assessments replace the continuous quantitative score, the model’s performance decreases to an AUC of 0.79, still significantly surpassing both the demographics and the system models. Importantly, the prediction models were evaluated on an unseen holdout set, providing a good estimate of their potential value when applied prospectively in clinical settings.

These results suggest that prediction models, based on routine developmental surveillance, can be used for ASD screening. Direct comparison of our prediction models with large studies^21,22,23^ that evaluated M-CHAT in a prospective cohort design showed that the sensitivity of the prediction models is higher than or equivalent to the sensitivity of M-CHAT at the same specificity.

Notably, in many of the studies^7^ that assessed M-CHAT, ASD screening and the criterion-standard evaluation were proximate in time. Consequently, their concordance was high, and so were the reported sensitivity and specificity. However, in studies that used prospective case confirmation from medical records, the sensitivity of M-CHAT was significantly lower.^7^ Among these studies, we compared our results with those that used large cohorts and long-term outcome follow-up.

The suggested prediction tool can be incorporated into the routine clinical workflow in several ways. In an environment that implements longitudinal developmental assessment, using age-appropriate milestones, the computation of the ASD score as per the compact model can be integrated into the EHR system. In a setting of screening during a single well-child visit, the snapshot model can be used. Additionally, milestone attainment measures can be continuous scores, such as the DSS, or simple binary indications that are independent of a specific developmental scale. The prediction of ASD requires defining a policy for setting the prediction age and the score’s thresholds, ensuring cost-effective performance.

### Limitations

The current study has several limitations. First, the ASD outcome was defined by an administrative source—eligibility of NII allowance. The exact timing of diagnosis was therefore unreliable, limiting the analysis of the potential benefit for shortening time to diagnosis. Second, the NII practices were adjusted over time to ease the eligibility process. This approach may introduce a selection bias in earlier years compared with later years. Third, it is unclear whether differences in ASD prevalence among cultural subgroups reflect true differences or originate from cultural differences in awareness, social stigma, and accessibility to services. Such differences can introduce a selection bias in the ASD group, which may affect the sensitivity analysis. Fourth, the MCHC developmental assessment visits are scheduled according to the vaccination guidelines. Consequently, at age steps without mandatory vaccinations, the adherence decreases; therefore, the population may be biased toward children at higher risk of developmental delays. Fifth, comparison with M-CHAT was not done on the same cohort; the sensitivity and specificity of the suggested score were compared with other studies.

Concurrently with an accurate ASD screening, health care systems should offer effective interventions to positively screened children, allowing them to achieve their full developmental potential. Future research should prospectively assess the combined benefit of early screening and timely treatment to the children’s developmental outcome.

## Conclusions

This study’s findings suggest that with the use of prediction models, ASD screening can be seamlessly integrated into routine early childhood developmental surveillance. These models achieved good performance when applied to both longitudinal and single-visit data and showed additive predictive value beginning at 12 months of age. The suggested approach may assist children in receiving timely interventions and achieving their developmental potential.

## Notes

Supplement 1. **eMethods.** Extended Methods **eResults.** Extended Results **eTable 1.** Population Characteristics Stratified by Development and Evaluation Sets **eTable 2.** Sizes of Final Train and Test Sets **eTable 3.** Characteristics of the Evaluation Set Before and After Exclusions **eTable 4.** Detailed Performance Comparison of Models With Different Variables Sets **eTable 5.** Detailed Performance Comparison of Predictions at Different Ages **eTable 6.** Comparison to MCHAT Studies **eTable 7.** The Israeli Ministry of Health Protocol for Developmental Evaluation of Infants and Toddlers From Birth Up to the Age of Six Years **eFigure 1.** Model Calibration **eFigure 2.** Bivariate Analysis
eReferences

Supplement 2.
Data Sharing Statement
