# Supplementary material for: Early Prediction of Autistic Spectrum Disorder Using Developmental Surveillance Data
Source: JAMA Netw Open. 2024 Jan 10;7(1):e2351052. doi: 10.1001/jamanetworkopen.2023.51052 (PMC12282497; doi:10.1001/jamanetworkopen.2023.51052)
Supplement: Supplement 1. — eMethods. Extended Methods eResults. Extended Results eTable 1. Population Characteristics Stratified by Development and Evaluation Sets eTable 2. Sizes of Final Train and Test Sets eTable 3. Characteristics of the Evaluation Set Before and After Exclusions eTable 4. Detailed Performance Comparison of Models With Different Variables Sets eTable 5. Detailed Performance Comparison of Predictions at Different Ages eTable 6. Comparison to MCHAT Studies eTable 7. The Israeli Ministry of Health Protocol for Developmental Evaluation of Infants and Toddlers From Birth Up to the Age of Six Years eFigure 1. Model Calibration eFigure 2. Bivariate Analysis eReferences [file jamanetwopen-e2351052-s001.pdf]

Supplementary Online Content

Amit G, Bilu Y, Sudry T, et al. Early prediction of autistic spectrum disorder using developmental surveillance data. *JAMA Netw Open*. 2024;7(1):e2351052. doi:10.1001/jamanetworkopen.2023.51052

eMethods. Extended Methods

eResults. Extended Results

eTable 1. Population Characteristics Stratified by Development and Evaluation Sets

eTable 2. Sizes of Final Train and Test Sets

eTable 3. Characteristics of the Evaluation Set Before and After Exclusions

eTable 4. Detailed Performance Comparison of Models With Different Variables Sets

eTable 5. Detailed Performance Comparison of Predictions at Different Ages

eTable 6. Comparison to MCHAT Studies

eTable 7. The Israeli Ministry of Health Protocol for Developmental Evaluation of Infants and Toddlers From Birth Up to the Age of Six Years

eFigure 1. Model Calibration

eFigure 2. Bivariate Analysis

eReferences

This supplementary material has been provided by the authors to give readers additional information about their work.

eMethod. Extended Methods

The developmental surveillance score (DSS)

DSS is a method for measuring milestone assessments and summarizing the overall developmental status by a continuous quantitative score. The methods for calculating it, as well as results demonstrating its validity, were reported in detail elsewhere[1]. Briefly, an attained milestone is assigned a score of 0, and an unattained milestone is assigned a positive number between 0 and 4, with higher values indicating a more severe concern, determined by the age of assessment of the population norms for attaining the milestone at that age (the greater the delay, the higher the score). For example, failure to attain a milestone at an older age than the attainment age of 95% of the population, was assigned a score between 3 and 4. For a set of milestones, either in a single visit or in multiple visits, DSS is defined as the average of the individual milestone attainment scores.

Feature extraction

For each child, various demographic and birth-related variables were taken at the index date. These included maternal age at birth, education, marital and employment status, child's sex, gestational age, birth type, newborn position, birth weight, birth head circumference, and Apgar scores at one and five minutes. Ethnicity-related variables such as cultural group and maternal country of origin were extracted for characterizing the study population, but were not used for training the prediction models, to avoid introducing social biases. Categorical covariates were transformed into multiple binary covariates using one-hot encoding. Missing values in categorical variables were handled as a distinct category.

For each visit within the appropriate covariate assessment window, all the assessed milestones and growth measurements were extracted, along with the variables recording the nurse's summary of the visit, parental concern regarding development and referrals for further evaluation. Milestone assessments were quantified using DSS and aggregated in two ways: by age step and by assessment age. In the per-step aggregation, all milestone scores for a given step in each domain were averaged. In a per-age aggregation all milestone scores for milestones evaluated during a given age range in each domain were averaged. Hence, the first aggregation method yielded variables like: "average score of language milestones of age step 12-18 months" (that is, the average score over all milestones associated with this age step), while the latter yielded variables like "average score of language milestones assessed at age 12-18 months" (that is, the average score over all milestones that were

© 2024 Amit G et al. *JAMA Network Open.*

evaluated at visits during which a child is at least 12 months old, but less than 18 months old). For example, a language milestone that belongs to the 12-18 months step but was evaluated at age 19 months would be included in the former, but not the latter variable, and vice versa for a language milestone of the 9-12 months step that was evaluated at age 13 months. For a simplified model, we considered variables derived from a binary score, which merely records the success or failure in milestone attainment, independent of the developmental scale.

Growth measurements included percentiles of weight, height and body BMI, according to the WHO growth charts [2]. Each type of growth variables was aggregated by the minimal and maximal values of all measurements within age windows of 6 months.

Missing values for continuous covariates were replaced by not-a-number (NaN) indicator. Children with such missing values were excluded from models' training and the main evaluation. However, as missing values can be handled by tree-based models, we evaluated the model's performance on this subgroup of children, which emulates missing a milestone assessment or an entire visit.

Importantly, classification models are designed to predict ASD likelihood at a specific age, and so only information that was available before the prediction age was used for training and evaluating the models.

Training of prediction models

The dataset was partitioned at random into a development set (80% of the children) and a holdout evaluation set (20% of the children), both with the same prevalence of ASD outcome. The development set was used for training, feature selection and hyperparameter tuning, by applying 10-fold cross validation. Once models were finalized, they were fitted on the whole development set and tested on the evaluation set. The output of a model is, for each child, a predicted probability for ASD outcome, which we interpret as an ASD score.

To model the data, we used Extreme Gradient Boosting (XGBoost), a decision tree based ensemble machine learning algorithm[3], which is commonly used for tabular data. The model's hyperparameters were determined by performing a grid search that greedily found the parameter values that maximized the area under the receiver-operating characteristic (ROC) curve (AUC) on the training set, by means of cross validation.

© 2024 Amit G et al. *JAMA Network Open.*

Sensitivity analysis

We evaluated the prediction performance on several subgroups, stratified by demographic, birth and clinical summary variables. These included sex, cultural groups, gestational age and indicators of concern raised by the parents or nurse. Additionally, we analyzed the subgroup of children excluded due to missed visits. For each subgroup we calculated the AUC and the sensitivity at a specificity of 95%, determined by a threshold on the group-specific ROC. We also computed the sensitivity and specificity of the model when using a global threshold, determined from the entire evaluation set.

eResults. Extended Results

Bivariate analysis

eFigure 2 depicts the crude odds ratio (OR) of the ASD outcome for selected demographic, birth, and milestone attainment variables, as well as for variables representing the clinical process. Indications from the clinical process, such as concern raised for the child's development, referrals to pediatrician or developmental tracking were associated with 3.3-3.4 times greater odds for an ASD outcome, compared to children without the analyzed indication. Consistent with Table 1, some demographic variables were associated with higher odds of ASD: male sex (OR 3.0, 95% CI [2.9,3.1]), maternal age over 40 years old (OR 1.6 [1.4,1.7]) and maternal high school education (OR 1.25 [1.2,1.3]). Failure at attaining milestones was also associated with higher ASD odds (eFigure 2b), even as early as at ages 1-3 months. From ages 9-12 month and older, ORs in most domains are significantly greater than 2. Failure at a milestone of any domain for age step 18-24 months was associated with high ORs between 3.2 and 4.1. The single variable with the highest odds ratio was failure at attaining social milestones at age step 12-18 months (OR 5.5 [5.0,6.1]).

eFigure 2d depicts the crude odds ratio of an ASD outcome per gestational age at birth, relative to 40 weeks. The odds ratio is higher for children born before week 40, and it increases as the pregnancy length is shorter. Interestingly, the odds ratio also increases for children born at gestational age of 41 and 42 weeks, with the latter significantly higher than one.

The results of the bivariate analysis are consistent with previous work on ASD risk factors. For example, identified indicators of ASD in early childhood include reduced frequency of social communication behaviors such as response to name, gaze to face, smile, vocalization and word verbalization by 12

© 2024 Amit G et al. *JAMA Network Open.*

months of age [4,5]. These indicators are represented in the milestones assessed in Israeli MCHCs, and the results of this study confirm the substantial contribution of features derived from attainment of language and social milestones to the likelihood of ASD diagnosis. Importantly, crude odds-ratios are oblivious to interaction between variables, and hence are limited in their ability to accurately measure the magnitude of effect. They are presented here to qualitatively reflect which variables are potentially relevant for the prediction.

© 2024 Amit G et al. *JAMA Network Open.*

Supplementary tables

eTable 1: Population characteristics stratified by development and evaluation sets.

Cohort characteristics		Development set			Evaluation set		
		All	No ASD	ASD	All	No ASD	ASD
n		949,917	935,193	14,724 (1.55)	237,480	233,799	3,681 (1.55)
Child sex, n (%)	Female	461410 (48.6)	457883 (49.0)	3527 (24.0)	115399 (48.6)	114519 (49.0)	880 (23.9)
	Male	488507 (51.4)	477310 (51.0)	11197 (76.0)	122081 (51.4)	119280 (51.0)	2801 (76.1)
Ethnic group, n (%)	Jewish	555205 (58.4)	545529 (58.3)	9676 (65.7)	139255 (58.6)	136825 (58.5)	2430 (66.0)
	Muslim Arab	184206 (19.4)	182949 (19.6)	1257 (8.5)	45577 (19.2)	45234 (19.3)	343 (9.3)
	Missing	148613 (15.6)	145802 (15.6)	2811 (19.1)	37253 (15.7)	36571 (15.6)	682 (18.5)
	Other	29630 (3.1)	28767 (3.1)	863 (5.7)	7443 (3.1)	7248 (3.1)	195 (5.2)
	Muslim Bedouin	16520 (1.7)	16485 (1.8)	35 (0.2)	4122 (1.7)	4109 (1.8)	13 (0.4)
	Druse	15743 (1.7)	15661 (1.7)	82 (0.6)	3830 (1.6)	3812 (1.6)	18 (0.5)
Mother birth country, n (%)	Israel	735560 (77.4)	725736 (77.6)	9824 (66.7)	183879 (77.4)	181379 (77.6)	2500 (67.9)
	Former Soviet Union	51073 (5.4)	49229 (5.3)	1844 (12.5)	12484 (5.3)	12052 (5.2)	432 (11.7)
	Missing	123182 (13.0)	120887 (12.9)	2295 (15.6)	30883 (13.0)	30318 (13.0)	565 (15.3)
	Europe	16267 (1.7)	16083 (1.7)	184 (1.2)	4065 (1.7)	4032 (1.7)	33 (0.9)
	Ethiopia	13325 (1.4)	12861 (1.4)	464 (3.2)	3475 (1.5)	3348 (1.4)	127 (3.5)
	North America	10510 (1.1)	10397 (1.1)	113 (0.8)	2694 (1.1)	2670 (1.1)	24 (0.7)
Employment status, n (%)	Missing	311171 (32.8)	305774 (32.7)	5397 (36.7)	77929 (32.8)	76581 (32.8)	1348 (36.6)
	Not Working	183208 (19.3)	180659 (19.3)	2549 (17.3)	45725 (19.3)	45027 (19.3)	698 (19.0)
	Student	38682 (4.1)	38339 (4.1)	343 (2.3)	9568 (4.0)	9485 (4.1)	83 (2.3)
	Working	416856 (43.9)	410421 (43.9)	6435 (43.7)	104258 (43.9)	102706 (43.9)	1552 (42.2)
Mother's education level, n (%)	Academic	272388 (28.7)	268732 (28.7)	3656 (24.8)	68092 (28.7)	67166 (28.7)	926 (25.2)
	Elementary	18864 (2.0)	18641 (2.0)	223 (1.5)	4719 (2.0)	4648 (2.0)	71 (1.9)

© 2024 Amit G et al. *JAMA Network Open.*

	High School	234532 (24.7)	230305 (24.6)	4227 (28.7)	58058 (24.4)	56996 (24.4)	1062 (28.9)
	Missing	329128 (34.6)	323750 (34.6)	5378 (36.5)	82637 (34.8)	81302 (34.8)	1335 (36.3)
	Tertiary Education	95005 (10.0)	93765 (10.0)	1240 (8.4)	23974 (10.1)	23687 (10.1)	287 (7.8)
Mother's family status, n (%)	Divorced	9320 (1.0)	8934 (1.0)	386 (2.6)	2316 (1.0)	2218 (0.9)	98 (2.7)
	Married	782734 (82.4)	771752 (82.5)	10982 (74.6)	195237 (82.2)	192508 (82.3)	2729 (74.1)
	Missing	116972 (12.3)	114955 (12.3)	2017 (13.7)	29407 (12.4)	28904 (12.4)	503 (13.7)
	Other	40441 (4.3)	39112 (4.2)	1329 (9.0)	10390 (4.4)	10044 (4.3)	346 (9.4)
	Widower	450 (0.0)	440 (0.0)	10 (0.1)	130 (0.1)	125 (0.1)	5 (0.1)
Consanguinity, n (%)	Missing	627759 (66.1)	616898 (66.0)	10861 (73.8)	157194 (66.2)	154468 (66.1)	2726 (74.1)
	No	268591 (28.3)	265100 (28.3)	3491 (23.7)	67215 (28.3)	66357 (28.4)	858 (23.3)
	Yes	53567 (5.6)	53195 (5.7)	372 (2.5)	13071 (5.5)	12974 (5.5)	97 (2.6)
Pregnancy wk, med [Q1,Q3]		39.3 [38.3,40.2]	39.3 [38.3,40.2]	39.1 [38.0,40.1]	39.3 [38.3,40.2]	39.3 [38.3,40.2]	39.0 [38.0,40.1]
Birth weight, mean (SD)		3.2 (0.5)	3.2 (0.5)	3.2 (0.6)	3.2 (0.5)	3.2 (0.5)	3.2 (0.6)
Head circum. (SD)		34.2 (1.6)	34.2 (1.6)	34.1 (1.9)	34.2 (1.6)	34.2 (1.6)	34.2 (1.8)
Apgar score one minute, n (%)	<8	37065 (3.9)	36193 (3.9)	872 (5.9)	9199 (3.9)	8973 (3.8)	226 (6.1)
	>=8	870776 (91.7)	857606 (91.7)	13170 (89.4)	217624 (91.6)	214351 (91.7)	3273 (88.9)
	Missing	42076 (4.4)	41394 (4.4)	682 (4.6)	10657 (4.5)	10475 (4.5)	182 (4.9)
Apgar score five minutes, n (%)	<8	8238 (0.9)	8020 (0.9)	218 (1.5)	2032 (0.9)	1981 (0.8)	51 (1.4)
	>=8	893428 (94.1)	879674 (94.1)	13754 (93.4)	223337 (94.0)	219911 (94.1)	3426 (93.1)
	Missing	48251 (5.1)	47499 (5.1)	752 (5.1)	12111 (5.1)	11907 (5.1)	204 (5.5)
Type of birth, n (%)	Caesarean section	165374 (17.4)	161658 (17.3)	3716 (25.2)	41414 (17.4)	40485 (17.3)	929 (25.2)
	Instrumental	48417 (5.1)	47547 (5.1)	870 (5.9)	12297 (5.2)	12076 (5.2)	221 (6.0)
	Missing	59061 (6.2)	57968 (6.2)	1093 (7.4)	14699 (6.2)	14418 (6.2)	281 (7.6)
	Spontaneous	677065 (71.3)	668020 (71.4)	9045 (61.4)	169070 (71.2)	166820 (71.4)	2250 (61.1)
	Breech	30727 (3.2)	30023 (3.2)	704 (4.8)	7463 (3.1)	7316 (3.1)	147 (4.0)

© 2024 Amit G et al. *JAMA Network Open.*

Newborn position, n (%)	Head	772251 (81.3)	761084 (81.4)	11167 (75.8)	193367 (81.4)	190549 (81.5)	2818 (76.6)
	Missing	134861 (14.2)	132241 (14.1)	2620 (17.8)	33638 (14.2)	32975 (14.1)	663 (18.0)
	Other	12078 (1.3)	11845 (1.3)	233 (1.6)	3012 (1.3)	2959 (1.3)	53 (1.4)
Mother's age, n (%)	<=20	33660 (3.5)	33239 (3.6)	421 (2.9)	8454 (3.6)	8324 (3.6)	130 (3.5)
	>20, <=40	823145 (86.7)	810777 (86.7)	12368 (84.0)	205800 (86.7)	202725 (86.7)	3075 (83.5)
	>40	28931 (3.0)	28219 (3.0)	712 (4.8)	7122 (3.0)	6933 (3.0)	189 (5.1)
	Missing	64181 (6.8)	62958 (6.7)	1223 (8.3)	16104 (6.8)	15817 (6.8)	287 (7.8)

eTable 2: Sizes of final train and test sets

	Compact model		Snapshot-score model		Snapshot-binary model	
Prediction age	Train size	Test size	Train size	Test size	Train size	Test size
24-36m	205,445	51,178	419,525	104,860	419,525	104,860
18-24m	229,391	57,190	411,614	102,533	411,614	102,533
12-18m	270,058	67,617	472,953	118,269	472,953	118,269
9-12m	284,000	71,138	360,502	90,216	360,502	90,216
6-9m	395,513	99,044	424,057	106,081	424,057	106,081
0-6m	445,393	111,382	445,393	111,382	445,393	111,382
	Full model		System model		Demographics model	
24-36m	186,615	46,501	379,118	94,450	539,849	135,022
18-24m	207,158	51,672	379,118	94,450	539,849	135,022
12-18m	260,671	65,212	457,870	114,373	539,849	135,022
9-12m	283,702	71,049	503,730	125,894	539,849	135,022
6-9m	395,333	98,995	529,163	132,263	539,849	135,022
0-6m	445,181	111,326	523,769	130,943	539,849	135,022

© 2024 Amit G et al. *JAMA Network Open.*

eTable 3: Characteristics of the evaluation set before and after exclusions.

Cohort characteristics		Before exclusions	After exclusions
n		237,480	57,190
Child sex, n (%)	Female	115399 (48.6)	27837 (48.7)
	Male	122081 (51.4)	29353 (51.3)
Ethnic group, n (%)	Jewish	139255 (58.6)	28883 (50.5)
	Muslim Arab	45577 (19.2)	15260 (26.7)
	Missing	37253 (15.7)	8275 (14.5)
	Other	7443 (3.1)	2079 (3.6)
	Muslim Bedouin	4122 (1.7)	1469 (2.6)
	Druse	3830 (1.6)	1224 (2.1)
Mother birth country, n (%)	Israel	183879 (77.4)	45065 (78.8)
	Former Soviet Union	12484 (5.3)	3773 (6.6)
	Missing	30883 (13.0)	6474 (11.3)
	Europe	4065 (1.7)	946 (1.7)
	Ethiopia	3475 (1.5)	594 (1.0)
	North America	2694 (1.1)	338 (0.6)
Employment status, n (%)	Missing	77929 (32.8)	18087 (31.6)
	Not Working	45725 (19.3)	12665 (22.1)
	Student	9568 (4.0)	2336 (4.1)
	Working	104258 (43.9)	24102 (42.1)
Mother's education level, n (%)	Academic	68092 (28.7)	19197 (33.6)
	Elementary	4719 (2.0)	1247 (2.2)
	High School	58058 (24.4)	16375 (28.6)
	Missing	82637 (34.8)	16044 (28.1)
	Tertiary Education	23974 (10.1)	4327 (7.6)
Mother's family status, n (%)	Divorced	2316 (1.0)	706 (1.2)
	Married	195237 (82.2)	48326 (84.5)
	Missing	29407 (12.4)	5470 (9.6)
	Other	10390 (4.4)	2641 (4.6)
	Widower	130 (0.1)	47 (0.1)
Consanguinity, n (%)	Missing	157194 (66.2)	42248 (73.9)
	No	67215 (28.3)	11056 (19.3)
	Yes	13071 (5.5)	3886 (6.8)
Pregnancy week, med [Q1,Q3]		39.3 [38.3,40.2]	39.3 [38.2,40.1]
Birth weight, mean (SD)		3.2 (0.5)	3.2 (0.5)
Head circum. (SD)		34.2 (1.6)	34.3 (1.5)
Apgar score one minute, n (%)	<8	9199 (3.9)	2134 (3.7)
	>=8	217624 (91.6)	54459 (95.2)
	Missing	10657 (4.5)	597 (1.0)
	<8	2032 (0.9)	419 (0.7)

© 2024 Amit G et al. *JAMA Network Open.*

Apgar score five minutes, n (%)	>=8	223337 (94.0)	55903 (97.7)
	Missing	12111 (5.1)	868 (1.5)
Type of birth, n (%)	Caesarean section	41414 (17.4)	10709 (18.7)
	Instrumental	12297 (5.2)	3397 (5.9)
	Missing	14699 (6.2)	3743 (6.5)
	Spontaneous	169070 (71.2)	39341 (68.8)
Newborn position, n (%)	Breech	7463 (3.1)	1987 (3.5)
	Head	193367 (81.4)	46722 (81.7)
	Missing	33638 (14.2)	7921 (13.9)
	Other	3012 (1.3)	560 (1.0)
Mother's age, n (%)	<=20	210543 (88.7)	51721 (90.4)
	>20, <=40	7122 (3.0)	1639 (2.9)
	>40	3711 (1.6)	1028 (1.8)
	Missing	16104 (6.8)	2802 (4.9)

© 2024 Amit G et al. *JAMA Network Open.*

eTable 4: Detailed performance comparison of models with different variables sets.

	Prediction at 18-24m			Prediction at 12-18m		
Model	AUC (CI)	Sensitivity at spec 95% (CI)	PPV at top 0.1% (CI)	AUC (CI)	Sensitivity at spec 95% (CI)	PPV at top 0.1% (CI)
Full	0.842* (0.830,0.853)	48.1 (45.3,50.8)	78.3 (66.6,88.2)	0.78* (0.77,0.79)	31.7 (29.5,34.2)	57.6 (44.6,69.2)
Compact	0.831* (0.821,0.842)	45.1 (42.6,47.5)	75.1 (63.2,86.0)	0.758* (0.747,0.769)	27.6 (25.6,29.7)	65.1 (52.2,76.1)
Snapshot-score	0.808* (0.799,0.818)	41.2* (39.2,43.2)	67.8 (58.8,76,5)	0.739* (0.730,0.749)	27.1* (25.5,28.6)	54.9 (45.8,63.6)
Snapshot-binary	0.792* (0.782,0.801)	34.4* (31.9,38.1)	41.8 (31.4,52.0)	0.724 (0.715,0.733)	23.0* (20.4,24.8)	30.4 (22.0,39.0)
System	0.776* (0.766,0.786)	32.1* (30.0, 34.0)	37.5 (27.7,47.9)	0.724* (0.714,0.733)	21.6* (20.0,23.2)	15.8 (9.6,22.8)
Baseline	0.667 (0.658,0.676)	11.8 (10.7,12.9)	1.5 (0,3.7)	0.666 (0.657,0.676)	12.1 (11.0,13.3)	2.8 (0.7,6.0)

*P<0.001 vs. next line's model

eTable 5: Detailed performance comparison of predictions at different ages. Values are reported for the compact and the snapshot model, constructed using the information that was available before the indicated prediction age.

	Compact model			Snapshot-score model		
Prediction age	AUC (CI)	Sensitivity at specificity 95% (CI)	PPV at top 0.1% (CI)	AUC (CI)	Sensitivity at specificity 95% (CI)	PPV at top 0.1% (CI)
24-36m	0.874* (0.864,0.885)	54.6 (52.0,57.4)	82.0 (70.6,92.2)	0.832* (0.823,0.842)	47.5 (45.6,49.7)	80.4 (73.1,87.5)
18-24m	0.831* (0.821,0.842)	45.1† (42.6,47.5)	75.1 (63.2,86.0)	0.808* (0.799,0.818)	41.2 (39.2,43.2)	67.8 (58.8,76.5)
12-18m	0.758* (0.747,0.769)	27.6† (25.6,29.7)	65.1 (52.2,76.1)	0.739* (0.730,0.749)	27.1† (25.5,28.6)	54.9 (45.8,63.6)
9-12m	0.716* (0.706,0.728)	19.0† (17.1,20.9)	24.7 (15.6,35.2)	0.709* (0.699,0.718)	18.1† (16.6,19.7)	23.0 (14.4,32.2)
6-9m	0.675† (0.663,0.685)	13.4 (12.1,14.8)	12.6 (6.1,19.2)	0.669 (0.659,0.679)	12.9 (11.6,14.5)	15.3 (9.4,22.6)
0-6m	0.666 (0.657,0.675)	12.6 (11.3,13.9)	6.2 (1.8,11.7)	0.666 (0.657,0.675)	12.6 (11.3,13.9)	6.2 (1.8,11.7)

*P<0.001 vs. next line's model; † P<0.01 vs. next line's model

© 2024 Amit G et al. *JAMA Network Open.*

eTable 6: Comparison to MCHAT studies. The compared prediction model was the 'compact' model applied at age 24 months on the evaluation set. The threshold of the predicted score was set to achieve the same specificity as the reference study, and the sensitivities were compared. P-values were calculated using a Chi square test.

Study	N	Screening subtype	Specificity	Sensitivity MCHAT	Sensitivity THIS-ASD 24m	P
**Carbone et al.**	26,364		97.8	33.1 (125/378)	33.7 (521/1545)	0.862
**Guthrie et al.**	20,375	Single	94.9	38.8 (176/454)	45.3 (700/1545)	0.016
		Repeated	93.7	49.6 (225/454)	49.4 (763/1545)	1.0
**Schjølberg et al.**	54,463	MCHAT20	94.9	27.6 (93/337)	45.3 (700/1545)	<0.001
		MCHAT23	92.5	31.2 (105/337)	53.0 (819/1545)	<0.001

eTable 7: The Israeli Ministry of Health's protocol for developmental evaluation of infants and toddlers from birth up to the age of six years. The protocol describes the evaluated milestones for each age group, the method of evaluation and the definition of which behavior qualifies as success.

milestone	Method of evaluation	pass
**Age 6 weeks to 3 months**		
	The baby lies on a flat surface, secure	
**Visually follows a moving object horizontally**	While lying on the back, the baby focuses his gaze on an object such as a colorful ring or a ball, the face of the parent or the examiner.<br><br>The object, the face of the examiner or the parent's should be within 20 to 30 centimeters from the baby's face.<br><br>Place the object or face in front of the baby's face and move in slow motion approximately 45 degrees to one side, back to the center, and then to the other side.<br><br>The test should not be accompanied by sounds or voices.	The baby visually follows a moving object 45 degrees horizontally in both directions
**Vocalizes in response to human voice**	Establishing eye contact with the baby is a preliminary condition to the accomplishment of this task.<br><br>It is recommended to guide the parent to make speech sounds to the baby, while providing a pause to allow the baby to listen and respond.	The baby makes sounds of joy or vocalize differently according to different needs.
**Smiles responsively**	Establishing eye contact with the baby is a preliminary condition to the accomplishment of this task.<br><br>The child's response to a stimulus sound or human face during the interaction should be examined.<br><br>To evoke a smile, the examiner can bring his face closer to the baby with a wide smile and speak softly.	The baby smiles in response to a human face or a human voice or stimuli.

© 2024 Amit G et al. *JAMA Network Open.*

**Raises head**	The baby lies on its abdomen. To see spontaneous movements, the examiner should refrain from placing its hand on the baby’s back or the pelvis.	The baby lifts his head off the surface momentarily and turns to either side.
**Age 3-6 months**		
**Visually follows a moving object vertically**	The baby follows a moving object vertically: up or down, within the middle line in a distance of 20 centimeters from his face	The baby follows a moving object vertically.
**Responds to rattling sound**	The baby lies down or is in its parents’ lap. A noise is produced by a rattle (a noisemaker, a knocking noise) at a distance of 50 centimeters from both sides of the baby's head and outside its vision range.<br><br>The noise production will be made in the same plane as the baby's ears and not higher.<br><br>Please note that there are no background noises during the evaluation.	The baby responds to the rattling sound in some manner, including: cessation of activity, eye blinking, head turning towards the sound origin.
**Responds to human presence**	When the baby is relaxed, a response to a human figure, touch or voice is examined.	The baby smiles spontaneously at a human figure, touch or human voice, or, when playing alone, stops crying when spoken to and sometimes mimics facial expressions.
**makes various sounds including constants (I.e. Mm rr gg)**	Various voices including consonants such as ahh or goo.<br>During the entire visit attention should be paid to the production of these various sounds.<br>The baby plays “vocally” by itself or, when played with, makes noises when it is excited or dissatisfied.	A variety of sounds are heard including consonants, such as – gg, grr, brr, mm, pp and etc.
**Hands together, manipulates fingers**	The action is observed while the baby lies on his back.<br><br>The disappearance of the initial congenital reflexes allows the baby to play with its hands in front of the center axis, in the midline.	The baby brings its hands to the midline, to his mouth or combines fingers.<br><br>Attention must be paid to the symmetry in hands movements to the midline while lying on the back.

© 2024 Amit G et al. *JAMA Network Open.*

**grasps an object**	During the examination the baby lies on its back. The task varies mildly according to the age at which the child arrives to the evaluation.	At the age 3-4 months the baby grasps an object that was placed in its palm, in each palm separately.<br><br>At the age of 4-5 months the baby reaches its hand to an object served to it at the middle line or placed on the chest and grasps with both hands.<br><br>At the age of 5-6 months the baby reaches its hand to objects that are within reachable distance, grabs them and approximates them to its body or looks at them.
**Head and chest up in prone position**	The baby lies on the abdomen, the examiner should refrain from intervening by placing hand on the baby's back or pelvis. The baby's movements are directed towards achieving a goal, such as a directed look at an object or figure. Their curiosity in exploring the environment should also be noted.	The baby lifts its head and chest while leaning on the forearms.
**Age 6-9 months**		
**transfers an object from one hand to the other**	The baby is lying on the back or sitting on the parent's lap, the examiner in front of it presents it with a toy in the middle line.	The baby reaches out one hand to the toy, grabs it and passes it to the other.<br><br>Watch the transfer in both directions and pay attention to the symmetry in the hand movements.
**makes repetitive syllables-constant or vowels**	Voice productions should be listened to throughout the visit.<br><br>During the "conversation" with the baby give time to allow it to listen and respond.	The baby makes mumbling sounds of syllables. The pronunciations are similar to the consonants and vowels of the language to which the baby is exposed, for example – mamha, dadha.
**rolls over from abdomen to back and back to abdomen**	Please notice that the roll over is from the right side to the left and from the left side to the right.<br><br>The examiner can interest the baby with a toy placed within an angle of 45 degrees above the head.	The baby rolls over from the back to the abdomen and from the abdomen to the back.
**Age 9-12 months**		

© 2024 Amit G et al. *JAMA Network Open.*

**taps two objects playfully**	The task should be performed when the baby lies on the back or sits on the parent’s lap. The examiner presents 2 toys or cubes, one for each hand.	The baby holds an object (cube) in each hand and taps them together.<br>The baby enjoys the sound and will continue to do so with pleasure.<br><br>Note symmetry in hands’ movements.
**crawls**	The child lies on its abdomen. The examiner should try to encourage the baby to move forward in a crawl.<br>Observe the style of crawling and the symmetry of activation of both sides of the body alternately.	The baby is observed crawling. All or some of the crawling styles are possible.
**vocalizes in a dialogue**	Voice productions should be listened to throughout the visit. During the "conversation" with the baby give a break to allow him to listen and respond.	The baby initiates and produces double syllables in a dialogue such as "aa-dada", parts of words, or alternatively makes sounds of animals.
**responds when addressed by name**	The mother or the nurse refers to the child by name (the name which the parents are accustomed of using) while being relaxed and in a soothing environment. The baby must be addressed once and then a response should be waited for. This could be repeated.	The baby responds when addressed by name. Responses can be varied, including cessation of activity, referral of the head or look, vocal response
**responds differently to familiar and stranger**	During the visit, the child's reactions to the familiar parent and to the examiner whom the child does not know should be monitored and evaluated.	There is a different reaction to the familiar parent, opposed to a certain reluctance to the unfamiliar character - the child will check, examine, or cry as a reaction to the examiner.
**feeds self**	Serve the child food to hold in its hand or a bottle	The baby feeds itself hand-held food, such as a cookie or a cooked vegetable.<br>The baby drinks from a bottle independently and from a cup with assistance.
**uses thumb-fingers grasp**	A small pea-sized object (such as a raisin or cereal) is served to the baby within a reachable distance, under observation.<br>Each hand should be evaluated separately.<br>The child should be prevented from bringing the object to the mouth.	The child holds a small object in its thumb and 2 fingers.<br>Later the child is able to grasp the object by the tips of the fingers between finger and thumb in a pinching motion.<br>Symmetry should be monitored.

© 2024 Amit G et al. *JAMA Network Open.*

**understands simple instructions**	Understanding instructions is evaluated by looking or pointing, such as: "Where is the light?", "Where is the bottle / cup?", "Applause", "wave hello", etc. without guidance or demonstration.<br>Ask the toddler to point or look at a familiar object in the room.	The baby follows three simple instructions, one of them must refer to a human figure, such as “where is mom\dad?”.
**gets to sit without support**	The child lies on its stomach or back. The child sits by itself from various positions.	The baby moves from a lying to a sitting position. Sitting can be in a variety of poses.
**Age 12-18 months**		
**says one word or pronounces meaningful sounds**	Encourage the baby to converse while giving time to allow it to listen and respond. It is important to listen to the baby during the entire visit.	The child says one word or pronounces meaningful sounds
**expresses will vocally or with gestures**	During the visit, the child's reactions to the familiar parent and to the examiner whom the child does not know should be monitored and evaluated.<br><br>The toddler can express desires in words, by pointing or by gesture. The toddler initiates.	The child initiates the expression of desires<br>vocally - uses words or parts of words.<br>By pointing - for example at a bottle - "drink", or a door - "walk";<br>Or with a gesture - for example, raises its hands to be picked up.
**makes eye contact and expresses reciprocity during joint game**	The toddler plays with the examiner or parent with a ball, car, dice, doll - according to its preferences.	The child focuses its gaze and follows both the object and the parent or examiner during the game.<br>There is reciprocity - the child reacts to the other and shares with the other while playing.
**walks with assistance**	The parent or examiner encourages walking by offering an interesting game at a suitable height and at a reasonable distance, when the child is assisted by furniture support (a stable chair, a stable stroller, etc.).	The toddler walks with support, moves between one furniture to the other, around walls and uses them for mobility, and / or with the help of an adult’s hand.
**points at familiar objects to request**	The toddler is asked to point to a familiar object in the room. For example: show me where the ball is.	The toddler understands and executes simple instructions with gestures, like pointing to an object upon request.
**pulls to stand**	The toddler should be encouraged to pull itself to a standing position with the help of	The toddler manages to rise from the floor with support in some manner, and sets itself up to a standing position.

	a chair or a low table, without an adult's assistance.	
**says 2-3 words**	Voice productions should be listened to throughout the visit.	The toddler consistently and regularly says 2-3 words including "dad" "mom" or pronounces consistent expressions understood to the environment and the family, such as: "Nana" for banana, "How" for dog.<br><br>In bilingual families, all words in each language must be referred to (for example, ball in both languages - is considered 2 words).
**Age 18-24 months**		
**climbs upstairs with assistance**	The child should be encouraged to climb stairs with a handrail that it can hold under supervision. the parent or examiner may also hold its hand in support.	The toddler goes up and down the stairs with the assistance of a helping hand or a handrail.
**walks without assistance**	The parent or examiner encourages walking by offering an interesting game at a suitable height and at a reasonable distance.	The toddler walks without assistance.
**eats independently with a spoon**	Information from the parent or primary caregiver should be provided regarding the manner of eating, the times and types of foods and textures; Toddler integration at family meals and experience of new foods.	The toddler eats independently with a spoon.
**familiar with at least one body part**	The toddler will be asked to show one or more body parts upon request and can show this on the parent or a doll.	The toddler recognizes at least one body part.
**builds a tower of cubes**	While sitting at a small table or on a carpet, the parent or examiner presents cubes to the toddler and asks it to build a tower. If the toddler does not perform the request, the parent or examiner will demonstrate building a tower.	At the age of 18 months - The toddler builds a tower from 3 cubes or more.<br>At the age of 21 months - The toddler builds a tower from 5 cubes or more.<br>Towards the age of two years - a tower from 6 cubes or more.<br>It should be noted that the toddler uses both hands during the tower building.

© 2024 Amit G et al. *JAMA Network Open.*

**has a vocabulary of over ten words**	The conversation between the parent and the toddler should be listened to at all times during the visit.<br>It is recommended to show the toddler a picture book and encourage it to talk, it is possible to use the parent's assistance.	The toddler has a vocabulary of over ten words.<br>You can get a report from the parent on the number of words or expressions which the toddler uses.
**squeezes and sticks out lips to give a kiss**	The parent or examiner asks the toddler to kiss the parent or a doll.	The toddler squeezes and sticks out lips to give a kiss.
**Age 2-3 years**		
**composes a sentence of at least two words**	The conversation between the parent and the toddler should be listened to at all times during the visit.<br>It is recommended to show the toddler a picture book and encourage it to talk, it is possible to use the parent's assistance.<br>It is possible to get a report from the parent regarding sentences which the toddler uses.	The toddler successfully combines two words into a sentence, for example - father-come, mother-water, want-water.<br>It is possible to get a report from the parent regarding sentences which the toddler uses.
**runs well without falling**	If possible, performed during the visit to the station. The examiner should pay attention to the child's organization before performing the task.<br>Multiple fails require attention and reference.	The toddler runs freely and confidently, stepping on each foot fully, while paying attention to obstacles in its path and its ability to bypass them without knocking them over.
**climbs up and down the stairs without an adult's assistance**	If possible, performed during the visit to the station. The child should be encouraged to go up and down the stairs with a railing that it can hold under supervision. Do not hold its hand for support.	The toddler goes up and down the stairs with the help of a railing, without the support of an adult.
**recognizes familiar objects and pronounces them by name**	The toddler is asked to name a variety of items or pictures that the examiner points to in the room while asking "What is it? What is it called?"	The toddler will name a number of items from the familiar environment, such as: clothing, furniture, games, fruits, animals and more.<br>Mispronunciations are acceptable.
**understands actions and speech without gestures**	The toddler is required to understand and perform actions described by the examiner, literally without making with a gesture or hint, with objects from the assessment kit.<br>For example:<br>Show me what do we drink with, or give me the cup.	The toddler understands at least 3 actions such as: give, show, put.<br>Understands at least one preposition such as - on, within.

**participates in a dialogue**	It is advisable to listen during the entire visit to the conversation that develops between the mother and the toddler. It should be monitored and seen whether a dialogue develops with the adults during the evaluation, whether the child expresses feelings and desires appropriately. Does it ask situation-appropriate questions like: who? what? where?.	The toddler initiates a verbal dialogue with the examiner or parent in a suitable situation, expresses its desires, responds toits interlocutor's response and is able to share experiences from everyday life.<br>Please note: Reverberating words without meaning or intention of communication will not be considered as speech aimed at communication purposes, and requires further evaluation.
**participates in daily activities**	Interview with the parent regarding: dressing, eating, bathing, urine and bladder control, playing.<br><br>A question about diaper weaning should be asked with great sensitivity according to the nurse’s acquaintance with the family, its customs and culture, and following the proper previous guidance.	
**imitates horizontal, vertical and circle lines**	The toddler sits on a chair at a small table next to the parent. The examiner or the parent hands the child a sheet of paper and a pencil, shows it how to draw horizontal, vertical and circle lines.	The toddler imitates horizontal, vertical and circle lines.
**Age 3-4 years**		
**expresses freely**	The examiner can use individual pictures or a book (descriptions of the child's daily life), and ask open-ended questions: what's going on here? or tell me or mom a story about what is happening in the picture - to allow the toddler to answer in a complete sentence.<br>The toddler is able to describe a number of pictures or different situations performed by the nurse.	The toddler's ability to describe pictures and combine words is evaluated.<br>The child performs successfully if there are two-word combinations, one of which is a verb, such as: child traveling, drinking milk, and also combinations such as: want to eat, or two-words combinations (which are not verbs), which are connected by a preposition such as: on, in. For example: a doll in a stroller, a bear in bed, a child on a bicycle, a doll in a chair.
**jumps from a stair**	The child is asked to jump off a step, independently - without support.<br>Up to 3 attempts should be allowed.<br>The action can be demonstrated.	The toddler jumps from a step and lands on both feet, independently - without support.

© 2024 Amit G et al. *JAMA Network Open.*

**puts on shoes and dresses independently without buttoning**	The examiner should ask if the child dresses by itself, independently.	The child dresses itself but needs help with buttoning and tying laces. Wearing only one item of clothing and shoes is sufficient.<br><br>The child should not be expected to differentiate between a right shoe and a left shoe.
**imitates patterns (+) and copies circles**	The child sits on a chair at a small table next to the parent. The examiner or the parent hands the child a sheet of paper and a pencil, the child is asked to draw a + pattern and an O pattern.	The child successfully draws a + or O pattern.
**Age 4-5 years**		
**stands up on one leg for 3 seconds**	The child is asked to stand on one leg. Standing should be on one leg without support with the raised leg free in the air. You can repeat the action up to 3 times. Initially the child will choose for itself on which leg to stand and then it should be encouraged to stand on the other leg. Differences between the quality of action on one leg compared to the other leg are expected.	The child stands up on one leg for 3-4 seconds.
**counts three cubes**	The examiner places cubes in front of the toddler and asks it to count them. Going forward the examiner extends their palm and asks to put 3 cubes in it. After the toddler completed the task, waiting, and counting along with him the number of cubes placed in the palm of the examiners hand. The toddler should associate each number with a cube.	The child counts at least three cubes.
**familiar with at least three prepositions**	It is advisable to place an object in a certain place so that the toddler will be able to understand and use the appropriate prepositions accordingly. For example: put a cube underneath the table. You can use pictures / book.	The child is familiar with at least 3 prepositions such as: on top, next to, underneath and more.
**plays with peer group**	Parental report regarding playing with peer group.	The child plays in a joint game with other children, initiates and develops imagination and imitation games. Is able to concentrate on board games. Familiar with simple rules of a game.

© 2024 Amit G et al. *JAMA Network Open.*

**dresses independently**	When preparing for a weight and height test check if the toddler is capable to undress and dress by itself. The ability to fasten buttons and zipper can be evaluated with the appropriate item in the development kit. Demonstration is possible if necessary.	The child is able to undress and dress itself, including button fastening.
**understandable speech**	During the visit at the station, listen to the developing conversation between the parent and the toddler: the toddler is able to tell a story, repeat a recitation / song, knows its name, uses male / female correctly.	The speech is clear and understandable to everyone.
**Age 5-6 years**		
**uses correct verbs and tense to describe a picture**	A book or pictures (including personal and family pictures) can be used to encourage the toddler to tell a story around the picture, or describe a simple story in sequence from pictures. In addition, you can evaluate the language skills during a free conversation with the toddler, by listening to it during the visit.	The child describes a picture using correct verbs and tenses.
**answers orientation questions such as name or age**	Ask the child to say its full name, age and address.	The child says its full name, age and address.
**jumps on one leg**	The child is required to jump 2-3 times on one leg in a row, out of 2-3 attempts. Demonstration is possible if necessary. An attempt to jump on the other leg should be evaluated, although better control on one leg is expected.	The child is required to jump 2-3 times on one leg in a row, out of 2-3 attempts.
**walks heel to toe**	The child is asked to walk in a straight-line heel to toe (tandem gait), 4-7 steps in 2 out of 3 attempts. Demonstration is possible.	The child walks in a straight-line heel to toe, 4-7 steps in 2 out of 3 attempts.
**draws a human figure**	The child sits on a chair at a small table next to the parent. The examiner or the parent hands the child a sheet of paper and a pencil, the child is asked to draw a human figure. Do not guide the child while performing the task to notice and complete missing parts (organs) in the drawing.	The child draws a human figure with at least 6 clear and understandable organs.
**copies geometrical shapes such as X and triangle**	The child sits on a chair at a small table next to the parent. The examiner or the parent hands the child a sheet of paper and a pencil. The examiner or parent shows the child a drawing of an X and a triangle and asks the child to copy the shapes.	The child copies an X or a triangle.

© 2024 Amit G et al. *JAMA Network Open.*

© 2024 Amit G et al. *JAMA Network Open.*

Supplementary figures

eFigure 1: Model calibration

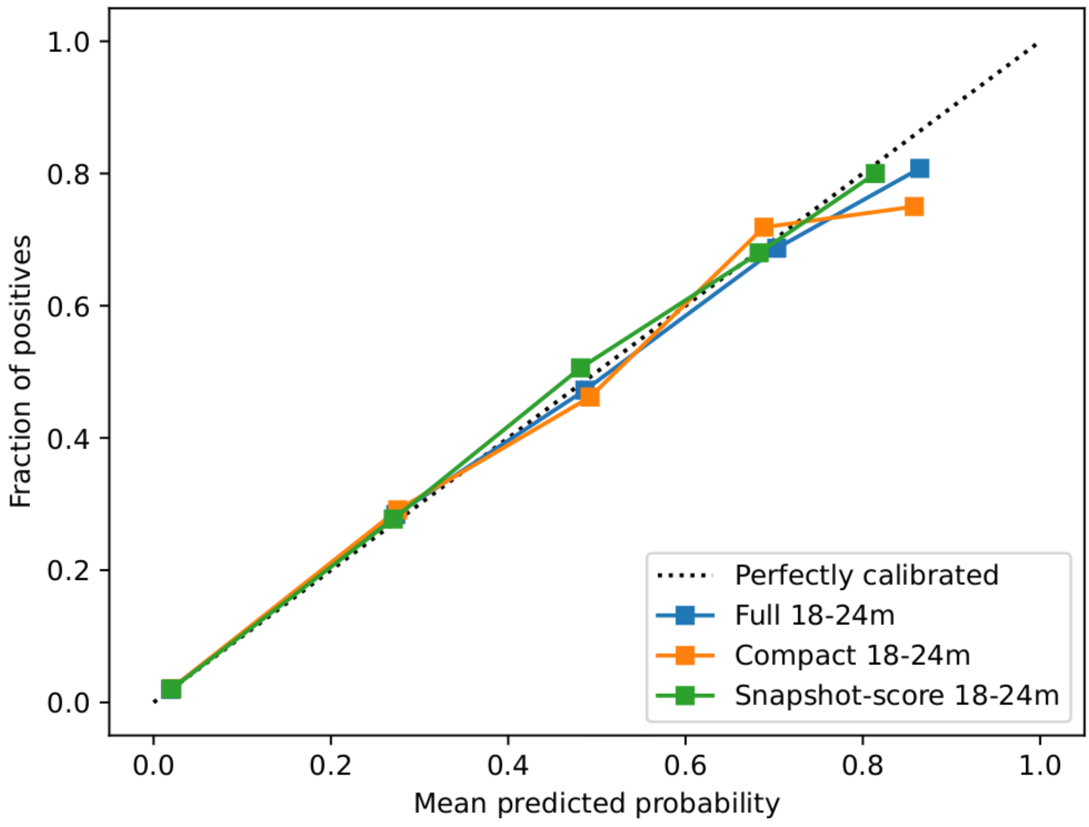

© 2024 Amit G et al. *JAMA Network Open.*

eFigure 2: Bivariate analysis

(a)

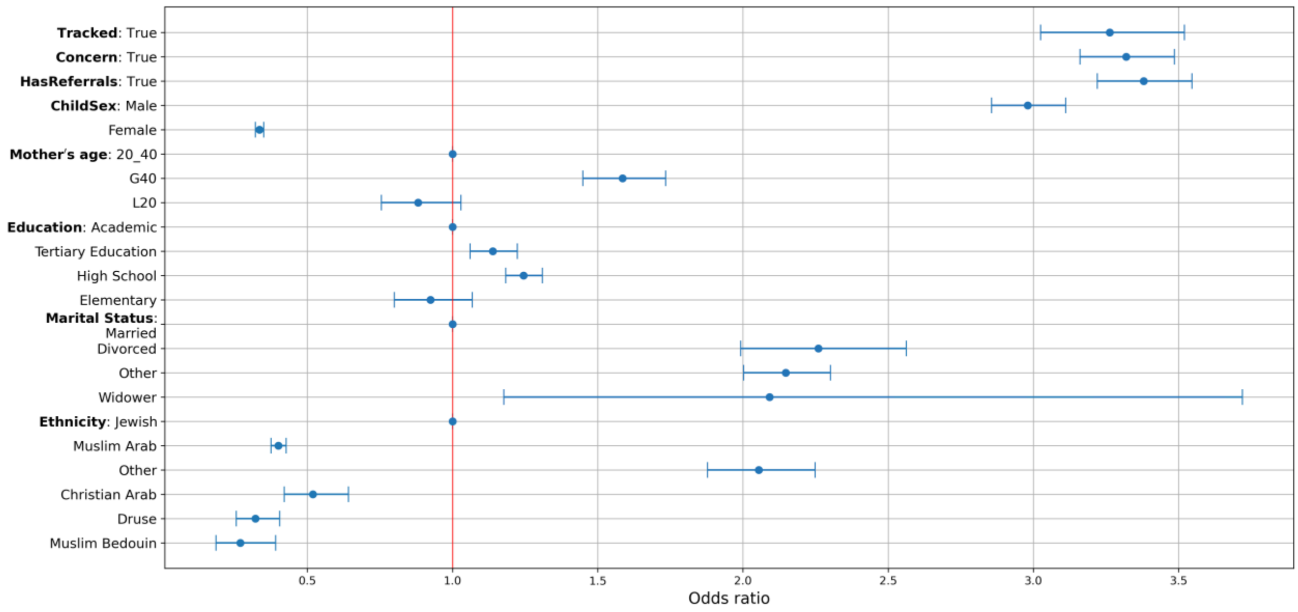

(b)

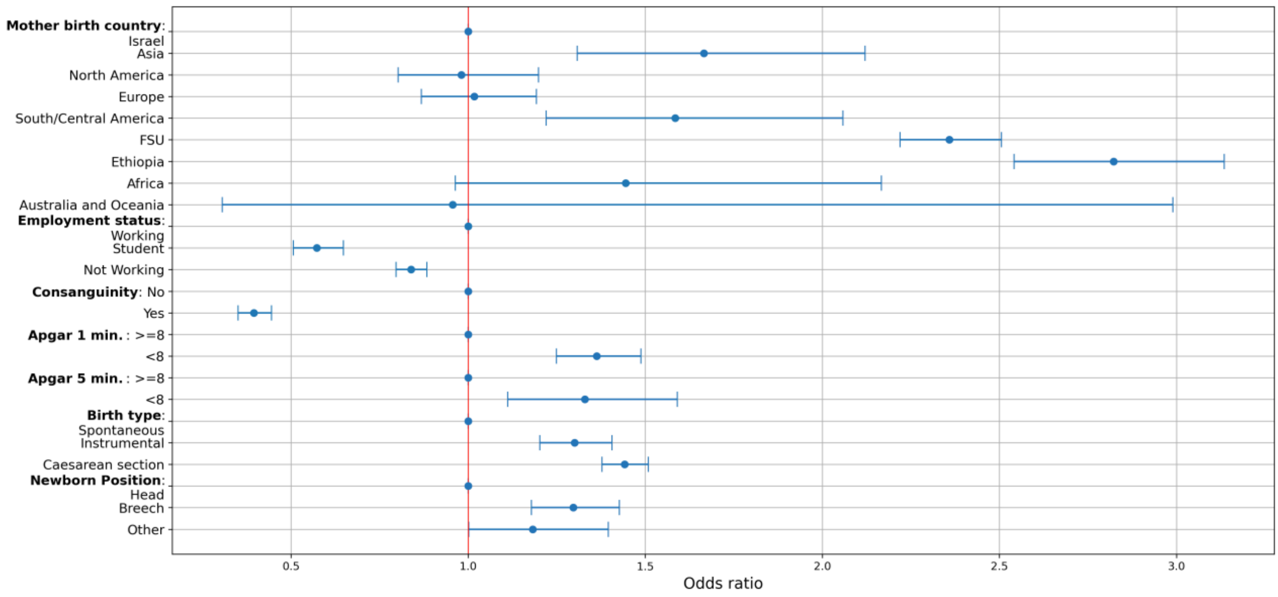

© 2024 Amit G et al. *JAMA Network Open.*

(c)

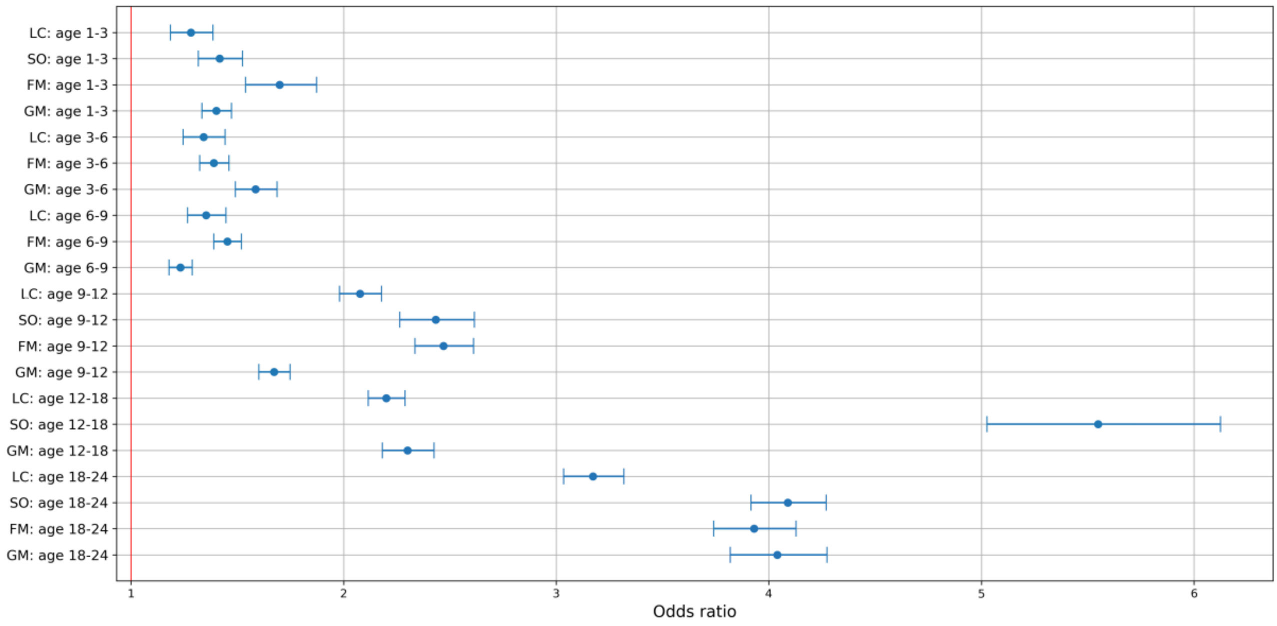

(d)

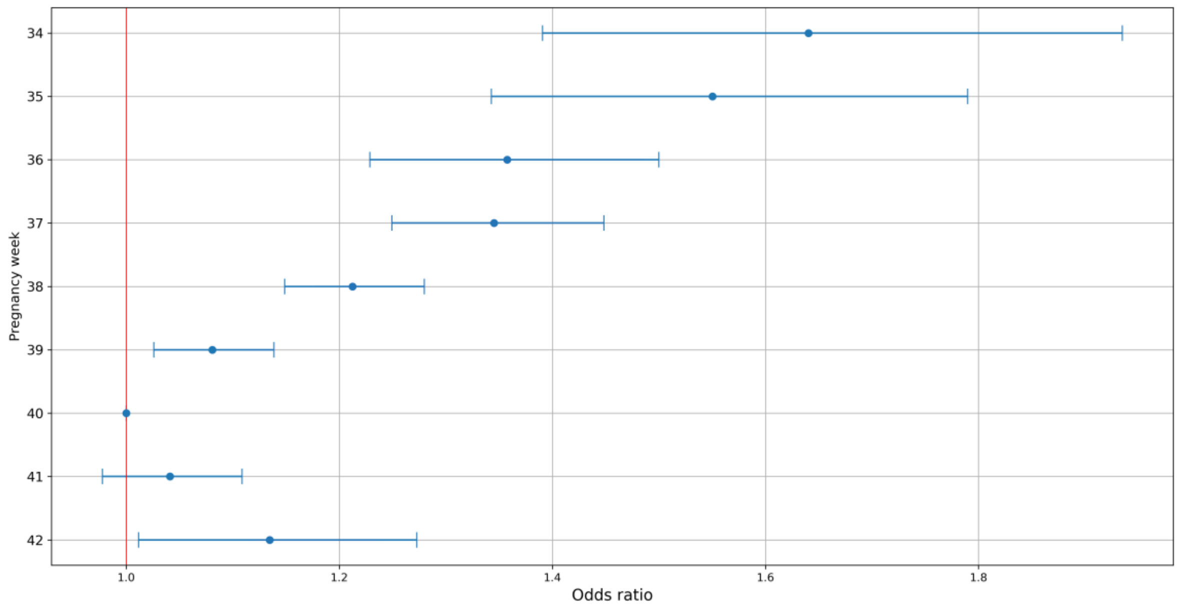

eFigure 2: Crude odds-ratios of an ASD outcome for variables describing (a) demographics and concern by the clinical system (a, b), failure at some milestone from the listed domain and at the listed age step (c), and gestational age (d). For categorical (non-boolean) variables, odds ratios are computed relative to the most common value. LC – language and communication; SO – social; FM – fine motor; GM – gross motor. Ages are indicated in months.

© 2024 Amit G et al. *JAMA Network Open.*

eReferences

1. Bilu Y, Amit G, Sudry T, et al. A developmental surveillance score for quantitative monitoring of early childhood milestone attainment: Algorithm development and validation. *JMIR Public Health Surveill*. Published online July 25, 2023. doi:10.2196/47315

2. WHO child growth standards: length/height-for-age, weight-for-age, weight-for-length, weight-for-height and body mass index-for-age: methods and development. Accessed July 26, 2023. https://www.who.int/publications-detail-redirect/924154693X

3. Chen T, Guestrin C. XGBoost: A Scalable Tree Boosting System. In: *Proceedings of the 22nd ACM SIGKDD International Conference on Knowledge Discovery and Data Mining*. KDD '16. Association for Computing Machinery; 2016:785-794. doi:10.1145/2939672.2939785

4. Ozonoff S, Iosif AM, Baguio F, et al. A Prospective Study of the Emergence of Early Behavioral Signs of Autism. *J Am Acad Child Adolesc Psychiatry*. 2010;49(3):256-66.e1-2.

5. Zwaigenbaum L, Bryson S, Rogers T, Roberts W, Brian J, Szatmari P. Behavioral manifestations of autism in the first year of life. *Int J Dev Neurosci*. 2005;23(2-3):143-152. doi:10.1016/j.ijdevneu.2004.05.001

© 2024 Amit G et al. *JAMA Network Open.*
