# Supplementary material for: Early Prediction of Autistic Spectrum Disorder Using Developmental Surveillance Data
Source: JAMA Netw Open. 2024 Jan 10;7(1):e2351052. doi: 10.1001/jamanetworkopen.2023.51052 (PMC12282497; doi:10.1001/jamanetworkopen.2023.51052)
Supplement: Supplement 2. — Data Sharing Statement [file jamanetwopen-e2351052-s002.pdf]

Data Sharing Statement

Amit. Early Prediction of Autistic Spectrum Disorder Using Developmental Surveillance Data. *JAMA Netw Open*. Published January 10, 2024. doi:10.1001/jamanetworkopen.2023.51052

Data
Data available: No

Additional Information
Explanation for why data not available: The de-identified patient-level data used for this study contains sensitive information and therefore is not available outside the secured research environment of the Israel Ministry of Health. Summary aggregate level data and analysis code for this study can be made available upon reasonable request to the corresponding author.
